# Gene and RNA Editing: Revolutionary Approaches to Treating Diseases

**DOI:** 10.1002/mco2.70389

**Published:** 2025-10-13

**Authors:** Jia‐Mei Li, Jie Huang, Yan Liao, Ting Hu, Chang‐Li Wang, Wang‐Zheqi Zhang, Chen‐Wei Huang

**Affiliations:** ^1^ Department of Neurology, Changhai Hospital Naval Medical University Shanghai China; ^2^ Department of Neurology The 971st Hospital of Navy Qingdao China; ^3^ Department of Anesthesiology, Changhai Hospital Naval Medical University Shanghai China; ^4^ Naval Medical University Shanghai China; ^5^ No. 940 Hospital of the Joint Logistics Support Force Lanzhou China; ^6^ Faculty of Psychology Naval Medical University Shanghai China

**Keywords:** CRISPR–Cas, gene editing, precision medicine, RNA editing

## Abstract

Gene editing and RNA editing technologies are advancing modern medicine by enabling precise manipulation of genetic information at the DNA and RNA levels, respectively. The third‐generation gene editing tools, particularly Clustered regularly interspaced shortpalindromic repeats (CRISPR)/CRISPR‐associated (Cas) system, have transformed genetic disease treatment with high efficiency, precision, and cost effectiveness, while RNA editing, via adenosine deaminase acting on RNA (ADAR) enzymes and CRISPR–Cas13, offers reversible regulation to avoid genomic integration risks. Despite advancements, challenges persist in delivery efficiency, tissue specificity, and long‐term safety, limiting their clinical translation. This review systematically discusses the molecular mechanisms and technological evolution of these tools, focusing on their promising applications in treating nervous system disorders (e.g., Alzheimer's, Parkinson's), immune diseases (e.g., severe combined immunodeficiency, lupus), and cancers. It compares their technical attributes, analyzes ethical and regulatory issues, and highlights synergies between the two technologies. By bridging basic research and clinical translation, this review provides critical insights for advancing precision medicine, reshaping disease diagnosis, prevention, and treatment paradigms.

## Graphical abstract

The image illustrates gene editing technologies: DNA editing using CRISPR–Cas9 and RNA editing via Cas13d, with their clinical applications and ethical risks. DNA editing allows precise gene modifications for conditions like amyotrophic lateral sclerosis [ALS] and Huntington's disease, while RNA editing supports multiplexed modifications. Ethical concerns involve gene function loss and germline editing controversies. Clinical applications include CAR‐T cell therapy for cancer and IL2RG gene knockout for immune reconstitution.

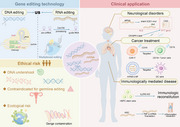

## 1 Introduction

Gene editing and RNA editing, as core technologies for the precise regulation of genetic information, intervene in biological systems at the DNA and RNA levels, respectively. Gene editing achieves targeted cleavage of DNA double strands using programmable nucleases (including CRISPR–Cas9, zinc finger nuclease [ZFN], and transcription activator‐like effector nuclease [TALEN]), harnessing cellular repair mechanisms to mediate gene knockout, insertion, or base substitution, providing a potential means to cure single‐gene genetic diseases like sickle cell anemia and Duchenne muscular dystrophy [1, 2, 3]. RNA editing dynamically alters RNA sequences through chemical modifications. For instance, in mammals, A‐to‐I (adenosine → inosine) editing mediated by ADAR enzymes (adenosine deaminases acting on RNA) regulates neurotransmitter receptor function. On the other hand, in plants, C‐to‐U (cytosine → uracil) editing directed by PPR (pentatricopeptide repeat) proteins contributes to environmental adaptability [4, 5, 6]. These two technologies complement each other in clinical applications: gene editing is superior in correcting DNA mutations at the source (as seen in the treatment of β‐thalassemia through hematopoietic stem‐cell therapy), while RNA editing, due to its reversible and transient regulatory characteristics, exhibits unique potential in the treatment of neurodegenerative diseases (e.g., ALS) and cancer immunotherapy [7, 8].

Gene editing technology has undergone three revolutionary stages: first, ZFNs first demonstrated the targeting ability of artificial nucleases in 1996. Second, TALENs enhanced programmability through modular design in 2010. Third, the CRISPR–Cas9 system, introduced in 2012, brought a revolution to the field by virtue of its simple design guided by single‐guide RNA (sgRNA) [9, 10, 11]. The subsequent development of high‐fidelity Cas9 variants, base editors, and prime editors further reduced the risk of off‐target effects. Additionally, these advancements expanded the scope of editing, enabling accurate single‐base modifications without causing double‐stranded breaks (DSBs) [12]. Since the discovery of mammalian A‐to‐I phenomenon in 1987, the field of RNA editing has gradually matured. This progress is attributed to the elucidation of the mechanism of ADAR enzymes in 1995 and the development of high‐throughput editing mapping in the 2010s. In 2017, the REPAIR (RNA editing for programmable A‐to‐I (G) replacement) system was launched. By using dCas13–ADAR fusion proteins, this system achieved precise RNA reprogramming in mammalian cells for the first time, laying a foundation for the development of new therapies for RNA‐related diseases such as Rett syndrome [13, 14]. In 2023, LEAPER (Leveraging Endogenous ADAR for Programmable Editing of RNA) 2.0 technology, which is based on a circular RNA (circRNA) self‐delivery system, boosted the in vivo editing efficiency to 90%. This milestone signifies the entry of RNA editing technology into a new stage of clinical translation [15].

At present, gene and RNA editing have progressed from basic research to the stage of clinical translation. However, challenges related to delivery efficiency, tissue specificity, and long‐term safety are impeding their broader application. This review bridges basic research and clinical practice by systematically analyzing these two technologies, focusing on their breakthroughs in treating nervous system disorders, immune diseases, and cancers. These fields represent high‐burden conditions with complex pathogenesis and limited therapies, perfectly showcasing the transformative potential of precision editing. They embody the complementary strengths of gene editing (permanent genomic correction) and RNA editing (reversible regulation), while serving as frontier areas where editing technologies have made notable breakthroughs, offering a focused lens to explore technical challenges and translational opportunities.

To explore these themes, we start by unpacking the core mechanisms and evolutionary paths of gene editing (e.g., CRISPR–Cas systems, base editors) and RNA editing (e.g., ADAR enzymes, CRISPR–Cas13), clarifying how each technology manipulates genetic information. Next, we delve into their therapeutic applications across three key disease areas: nervous system disorders (Alzheimer's disease [AD], Parkinson's disease [PD]), immune diseases (severe combined immunodeficiency [SCID], lupus), and cancers—detailing specific strategies, landmark studies, and current limitations. We then contrast their technical strengths and weaknesses, examine ethical and regulatory considerations, and discuss how their combination could amplify therapeutic potential. Finally, we synthesize these insights to outline future directions, emphasizing the need to address delivery and safety barriers to accelerate translation, ultimately advancing precision medicine from concept to clinical impact.

## 2 Overview of Gene Editing Technologies

Gene editing technology refers to the precise modification of an organism's genome through artificial means, with core processes including the identification of target genes and the cutting or modification of DNA. The emergence and development of this technology have significantly advanced biomedical research and agricultural science. Gene editing can be broadly categorized into four types based on different editing principles: nuclease‐mediated gene editing, recombinase‐mediated gene editing, base editing, and prime editing (PE).

### 2.1 Basic Principles of Gene Editing

**FIGURE 1:**
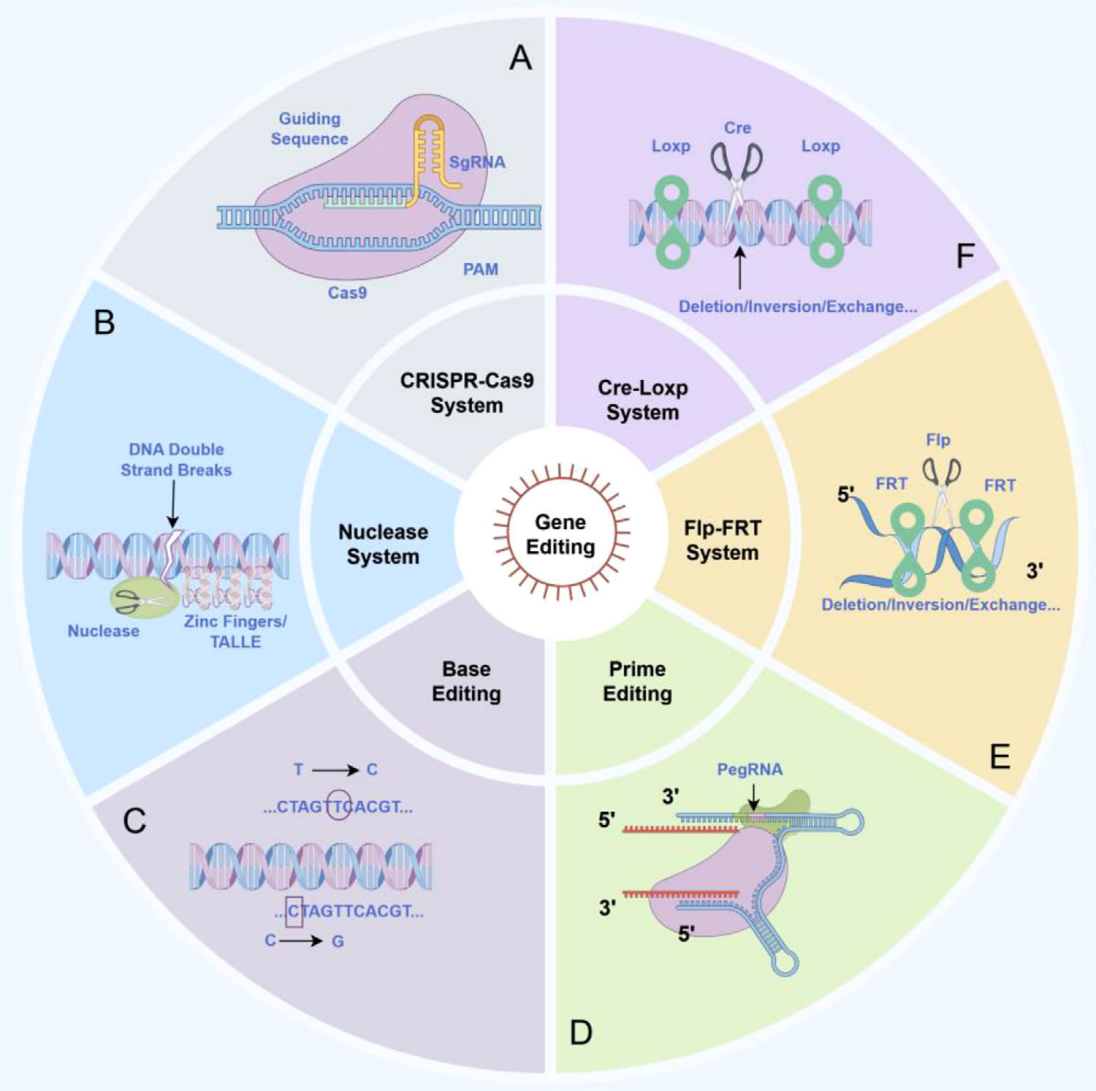
Schematic representation of gene editing technologies and their core mechanisms (by Figdraw). (A) CRISPR–Cas9: sgRNA guides Cas9 to induce DSBs, repaired via NHEJ/HDR. (B) Nuclease system (ZFN/TALEN): DNA‐binding domains direct FokI to cleave DNA. (C) Base editing: CBE/ABE mediates single‐base conversion without DSBs. (D) Prime editing: pegRNA guides Cas9 nickase/reverse transcriptase to write new sequences. (E) Flp–FRT: Flp recombinase mediates DNA deletion/inversion via FRT sites. (F) Cre–LoxP system: By inserting the LoxP site in advance, the Cre gene acts as a pair of scissors to cut out specific genes.

Gene editing technology refers to the technology that precisely modifies the genome of an organism through artificial means, and its core processes are the identification of the target gene and the cutting or modification of DNA [16]. The emergence and development of this technology have greatly promoted the development of biomedical research and agricultural science [17]. According to different editing principles, gene editing can be roughly divided into nuclease‐mediated gene editing, recombinase‐mediated gene editing, base editing, and PE. Nuclease‐mediated gene editing uses engineered nucleases to introduce DSBs at specific genomic locations [18]. The introduction of DSBs can lead to gene disruption or facilitate the insertion of new genetic material through the natural repair process of cells, such as nonhomologous end joining (NHEJ) or homologous directed repair (hDR) [19]. Precisely because it can trigger DSBs at specific positions, nucleic acids generate intense repair reactions while achieving precise editing. Nuclease‐mediated gene editing is widely used in a variety of organisms and is applicable to various gene editing [16]. In addition, different from nucleases, recombinase‐mediated gene editing refers to the use of recombinases to recognize specific DNA sequences and perform DNA recombination between these sites, thereby achieving gene knockout, insertion, inversion, or substitution [20]. Compared with nuclease‐mediated gene editing, recombinase‐mediated gene editing does not require nucleic acid cleavage, reducing specific risks [21]. In addition, before achieving recombinase‐mediated gene editing, specific sites need to be preinserted, which greatly reduces the off‐target effect, that is, cutting DNA at nontarget sites, leading to unexpected mutations [22]. In addition, base editing and PE are also common gene editing methods. The former can achieve precise conversion of single bases without introducing DSBs [23]. The latter uses a guide RNA (gRNA) called pegRNA (PE gRNA) to direct the editing process and can achieve any type of base editing at any part of the nucleic acid [24]. In summary, gene editing can be roughly divided into the following steps: the identification and modification of the target gene, as well as the necessary cell repair. Completing gene editing can control the expression and transcription of proteins, thus affecting various life processes of organisms. Figure 1 provides an overview of the basic principles of gene editing.

### 2.2 Gene Editing Tools

According to different editing principles, different types of gene editing require different tools. Nuclease‐mediated editing involves DNA cleavage followed by repair. A prominent example of a nuclease‐based tool is the CRISPR–Cas9 system. While its workflow can be intricate, its design is straightforward and cost‐effective. The CRISPR–Cas9 system is composed of gRNA and Cas9 nuclease. The gDNA can recognize the target DNA sequence through base pairing and guide the CRISPR nuclease to introduce DSB at specific sites [25]. In this system, the activity of the CRISPR enzyme depends on the sequence and structural characteristics of the gRNA, and the two interact with each other. Specific sequence characteristics, such as the protospacer and protospacer‐adjacent motif (PAM), are crucial for the effective cleavage of Cas9 [26]. Although the CRISPR–Cas9 system is widely applied, its disadvantages of a high off‐target rate and dependence on hDR are often criticized. In the nuclease system, TALEN and ZFN are also relatively common. However, due to the cumbersome assembly of these two, their degree of applicability is not as high as that of the CRISPR–Cas9 system [27]. TALEN and ZFN are composed of TALE proteins or zinc finger proteins and FokI nucleases. Like CRISPR–Cas9, these DNA‐binding domains recognize specific sequences, and the FokI nuclease generates DSBs at the target loci [28]; however, this complexity restricts their broader adoption relative to CRISPR–Cas9.

In recombinase‐mediated gene editing, the Cre–LoxP system, which avoids DSB, is widely used in gene editing due to its high precision and reversibility, especially in conditional knockout or gene activation of mouse tissues [29]. However, it is necessary to insert a LoxP site in the gene in advance, restricting its use to certain genetic backgrounds [30]. Studies have shown that by optimizing the spacer sequence of the Cre/LoxP system, the activity and specificity of the recombinase can be improved, thereby achieving more precise gene editing [31]. The Cre–LoxP system can also be combined with the CRISPR–Cas9 system to improve the activity of the recombinase and achieve more precise gene editing [31]. The Flp recombinase‐mediated Flp–FRT system is similar to the Cre–LoxP system. However, due to its lower efficiency than the Cre–LoxP system, it is more often used for gene recombination in Drosophila or plants [32]. Through continuous optimization and improvement, the application prospects of the Flp–FRT system in genome editing will be broader.

In base editing, the cytosine base editor (CBE) and adenine base editor (ABE) have been applied in practice [33]. As the name implies, the former converts the base C·G to G·A, while the latter converts A‐T to G‐C [34]. Since both carry out single‐base changes without causing DSB, commonly used to correct point mutations [35]. It is precisely because of this single base mutation that there may be bystander editing, resulting in the editing of adjacent bases as well [36]. PE is a gene editing method that combines Cas9 nickase with reverse transcriptase and directly writes new sequences through pegRNA. This method does not require DSB or donor DNA and can perform various types of editing [37]. However, its design is complex and its overall efficiency is lower than that of the standard CRISPR–Cas9 approach [38].

Gene technologies with different principles correspond to different gene editing tools. How to select appropriate gene editing tools requires comparing the different characteristics of various gene editing technologies and choosing the appropriate tools.

## 3 Overview of RNA Editing Technologies

RNA editing technology refers to the temporary and reversible modification of posttranscriptional RNA, achieved by altering the bases or sequences of RNA, distinguishing it from DNA editing. RNA editing is typically conducted using specific tools or enzymes, with naturally occurring ADAR and APOBEC enzymes (apolipoprotein B mRNA‐editing enzyme catalytic polypeptide) mediating A‐to‐I and C‐to‐U editing, respectively. Unlike DNA editing, RNA editing does not modify the genome, resulting in a rapid loss of editing effects upon RNA degradation, which makes it particularly suitable for short‐term or dynamic regulation. Methods of RNA editing can be categorized into several types: ADAR enzyme‐based editing systems, the CRISPR–Cas13 system, antisense oligonucleotide (ASO)‐mediated editing, and the emerging LEAPER technology. The significance of RNA editing technology lies in its unique properties of immediacy, reversibility, and simplicity, positioning it increasingly prominently in scientific research, especially within the field of genetic engineering. Furthermore, the presence of key enzymes in nature facilitates the expansion and optimization of existing technologies, paving the way for the development of more efficient RNA editing methods in the future.

### 3.1 The Basic Principles and Main Methods of RNA Editing

Unlike gene editing at the DNA level, RNA editing acts on the RNA level, making temporary and reversible edits by modifying the bases or changing the sequences of the transcribed RNA [39]. RNA editing modifies the RNA bases through specific tools or enzymes [40]. RNA editing also occurs naturally. For example, in mammals, ADAR enzymes can mediate A‐to‐I editing, and APOBEC enzymes can mediate C‐to‐U editing [41, 42]. In genetic engineering, artificial RNA editing has a broader application prospect, that is, precisely modifying RNA through the use of engineered tools [43]. Since RNA editing occurs posttranscriptionally and does not change the genome, the editing effect disappears rapidly when the RNA is degraded [14]. Moreover, RNA editing cleverly avoids the risk of chromosomal abnormalities caused by of DSB, making it particularly suitable for short‐term or dynamic regulation [44].

The main methods of RNA editing can be classified as: the editing system based on ADAR enzymes, the CRISPR–Cas13 system, the editing mediated by ASOs, and emerging technologies such as LEAPER. ADAR enzymes are enzymes catalyzing the conversion of A‐to‐I, and this process occurs in dsRNA (double‐stranded RNA). ADAR enzymes are crucial for transcriptome diversity and proper mammalian development [45]. By combining endogenous ADAR enzymes with engineered gRNAs, precise editing and regulation of specific RNA sites can be achieved. This editing method can not only change the base pairing properties of RNA but also modify codons, thereby affecting the function of proteins [46]. In addition, the editing activity of ADAR enzymes can prevent inappropriate innate immune activation by dsRNA, which is crucial for maintaining the normal function of cells [47]. ADAR enzymes can be combined with a variety of tools to enhance their functions and applications. For example, when combined with SNAP‐tag proteins, they can be directed toward the target RNA after chemical induction, thus achieving efficient RNA editing [48]. Through this combination, researchers can edit specific RNAs without affecting the stability of the genome. This method has potential application value in the treatment of diseases related to RNA editing [49]. In addition, ADAR enzymes can also be combined with other tools, such as the CRISPR/Cas system, to form a new RNA editing platform. These platforms utilize the editing ability of ADAR enzymes and combine the targeting characteristics of the CRISPR/Cas system to achieve precise editing of RNA. This combination not only improves the editing efficiency but also reduces the editing of nontarget sites, thereby increasing the specificity of editing, which is also known as RESCUE [50]. ADAR enzymes are endogenous enzymes with low immunogenicity, and editing endogenous RNA does not require the exogenous delivery of editing enzymes, which is a major advantage [51]. However, given that its editing efficiency is affected by the secondary structure of RNA, gRNA design must be carefully optimized [52]. Another key RNA editing tool is the CRISPR–Cas13 system. It uses the RNA targeting ability of the Cas13 protein and fuses deaminase enzymes to achieve RNA editing. There are multiple subtypes of the Cas13 protein, and especially Cas13d has attracted much attention due to its small structure and high specificity [52]. In this system, Cas13 recognizes the target RNA through crRNA without the involvement of tracrRNA [14]. Cas13‐mediated RNA editing has high specificity and can achieve multitarget editing. Moreover, Cas13 has the activity of “collateral cleavage,” which can increase the editing efficiency. Different from endogenous ADAR enzymes, researchers have conducted an immunological evaluation of Cas13d and found that most healthy donors have an immune response to Cas13d, which needs to be considered when developing Cas13d as a therapeutic tool [14]. In addition, the editing mediated by ASO is also a common type of RNA editing. Chemically modified ASOs are designed to bind complementarily to the target RNA and recruit endogenous ADAR enzymes, enabling precise RNA editing without disrupting the natural editing balance [44]. There are various ASO drugs, and most of them are easily delivered small molecules. However, precisely because of their size, multiple doses may be needed, making them less efficient than other systems [53]. Based on the above traditional technologies, researchers have integrated the advantages of various technologies, optimized their defects, and developed the LEAPER technology. This technology does not require the participation of exogenous proteins and can reduce the in vivo immune response. Its circRNA offers greater stability and resistance to degradation [54]. In the LEAPER technology, circRNA recruits endogenous adenosine deaminases to achieve RNA editing. Compared with traditional linear RNA, circRNA has greater in vivo and in vitro efficiency and specificity, which can effectively reduce the editing of nontarget sites [55].

**FIGURE 2:**
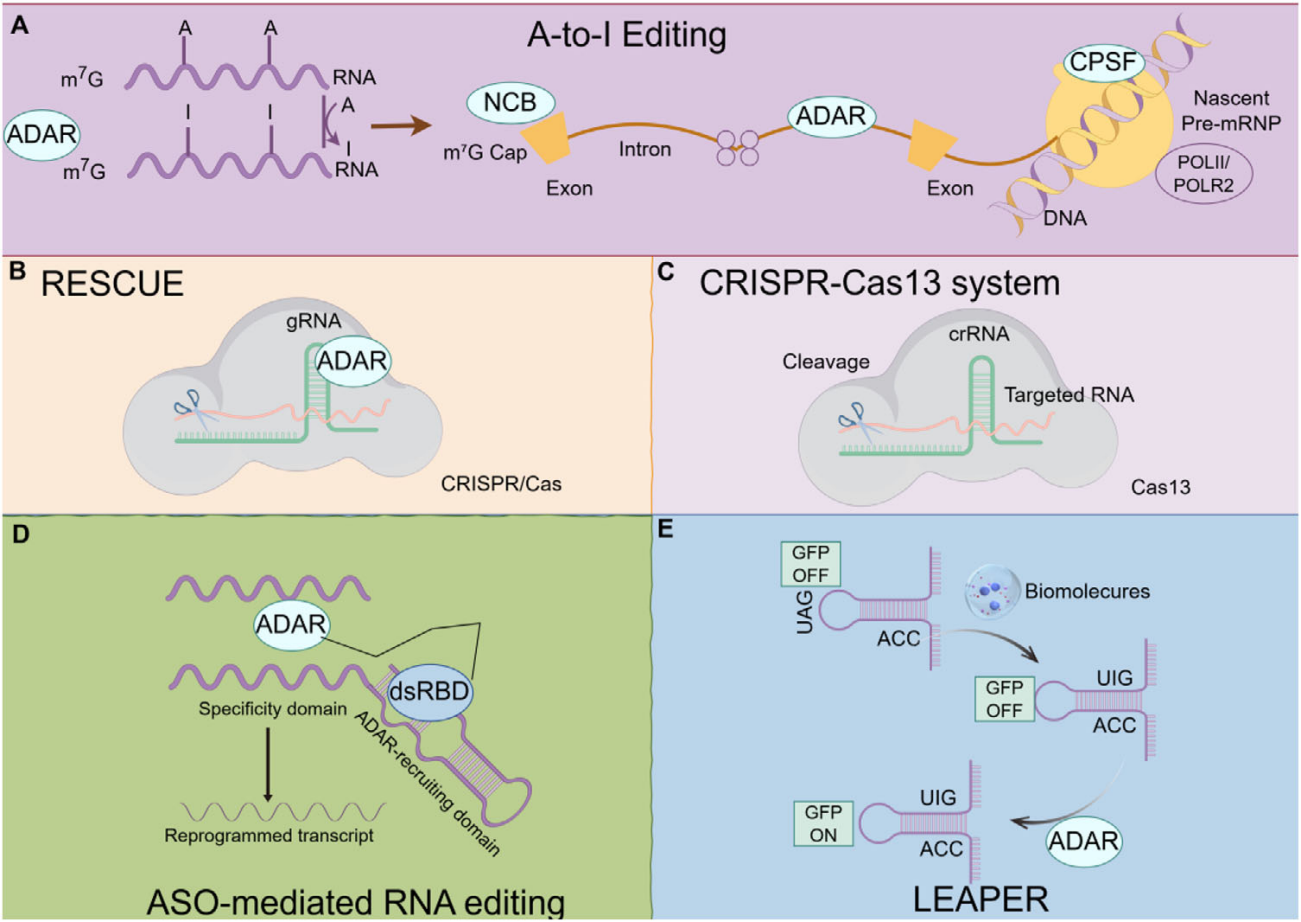
Schematic representation of RNA editing technologies and their core mechanisms (by Figdraw). (A) ADAR‐mediated A‐to‐I editing: ADAR converts A→I in dsRNA. (B) RESCUE: dCas13–ADAR fusion (guided by sgRNA) mediates C→U editing. (C) CRISPR–Cas13: Cas13 cleaves target RNA or fuses with deaminases for editing. (D) ASO‐mediated editing: ASOs recruit endogenous ADAR to specific RNA sites. (E) LEAPER: Circular guide RNAs recruit ADAR for A‐to‐I editing (enhanced stability vs. linear RNAs).

Therefore, due to its unique advantages of being instantaneous, reversible, and simple, RNA editing occupies an increasingly important position in scientific research and has a quite broad application prospect in genetic engineering. Moreover, due to the advantage that the key enzymes themselves exist in nature, it is easier to further expand and optimize emerging technologies on this basis, and more efficient RNA editing technologies will emerge in the future. Figure 2 provides an overview of the basic principles of RNA editing.

### 3.2 The Application Prospect of RNA Editing in Disease Treatment

Many diseases, especially genetic disorders, cancers, and neurodegenerative conditions, are caused by gene mutations [56]. Since the inception of genetics, scientists have been obsessed with modifying the genes of organisms to increase their productivity or eliminate diseases. Due to ethical concerns, the scientific community generally opposes experiments of gene editing in the human bodRNA editing occurs posttranscriptionally, and it is reversible and instantaneous. Currently, it is widely applied and safe in the research of various diseases. This characteristic endows RNA editing with great potential and advantages in the treatment of acute or stage‐specific diseases [57].

## 4 Applications of Gene Editing and RNA Editing in the Therapy of Nervous System Disorders

Neurological disorders are diseases marked by structural or functional abnormalities in the central or peripheral nervous system, resulting from genetic, infectious, traumatic, metabolic, degenerative, or immune‐related factors. Gene Editing or RNA Editing primarily targets central nervous system (CNS) disorders. This includes, but is not limited to, urgently addressed neurodegenerative diseases such as AD, Huntington's disease (HD), and PD, as well as neuronal injury disorders. In these neurodegenerative conditions, the predominant pathological features observed in patient brain tissue involve the deposition of abnormal proteins, notably amyloid‐β (Aβ) plaques, tau neurofibrillary tangles (NFTs), filamentous Lewy bodies, and α‐synuclein (α‐Syn) within dystrophic Lewy bodies [58, 59]. Gene editing and RNA editing techniques mitigate the risk of developing these diseases by targeting and modifying the genes responsible for producing these aberrant proteins. The etiology and pathogenesis of neurodegenerative neurological disorders are multifaceted, and consequently, therapeutic approaches must adapt dynamically [60]. Precise spatiotemporal control over the initiation and termination of editing activity represents a major focus of current research. In recent years, with advancements in medical technology and the accelerating pace of population aging, the morbidity and mortality rates associated with neurological disorders have been on the rise [61]. However, due to their complex pathogenesis and diverse clinical manifestations, these disorders pose significant challenges in clinical diagnosis, and conventional therapeutic approaches often have limited success [62]. Current clinical practice increasingly emphasizes precision and personalization in treatment. Gene editing and RNA editing, leveraging their inherent targeted nature, enable the development of patient‐specific organoids derived from the patient's own tissues and cells. This facilitates the creation of humanized, editable, and personalized pathological models, providing a highly suitable platform for clinical drug development. Against this backdrop, advanced strategies such as gene editing and RNA editing have emerged as promising avenues for the mechanistic dissection and intervention of neurological disorders [6, 63, 64].

### 4.1 Applications and Strategies of Gene Editing Therapy for Nervous System Disorders

The dominant gene editing technologies currently include ZFNs, TALENs, and CRISPR–Cas systems [65]. Both ZFNs and TALENs rely on protein–DNA binding domains to direct nucleases to cleave specific DNA sequences. Although they showed promise in early studies, their complex protein engineering design and high synthesis costs have limited clinical translation [65]. By contrast, the CRISPR–Cas system utilizes sgRNA to precisely target specific loci, substantially streamlining the gene editing workflow and significantly enhancing editing efficiency and technical scalability [66]. However, the CRISPR–Cas system still faces significant challenges: delivery efficiency is hindered by the blood–brain barrier, viral vectors pose immunogenicity, and payload limitations, and issues such as off‐target effects and long‐term safety concerns remain unresolved [67, 68, 69, 70].

#### 4.1.1 Alzheimer's Disease

AD is a neurodegenerative disorder characterized by clinical manifestations of dementia, cognitive impairment, and memory loss [71], with pathological hallmarks including Aβ42 deposition, toxic phosphorylated tau accumulation, and neuronal loss [72]. AD is classified into familial AD (FAD) and sporadic AD (SAD) forms. FAD is associated with genetic mutations, such as those in the amyloid precursor protein (APP), presenilin 1 (PSEN1), and presenilin 2 (PSEN2) genes, which lead to excessive production of Aβ42 [73]; SAD is linked to risk genes like apolipoprotein E4 (APOE4), whose carriage significantly increases AD susceptibility [74]. Currently, clinical management of AD offers limited and largely ineffective therapeutic options. Primary approaches rely on cholinesterase inhibitors for treating AD dementia or utilize memantine as an alternative therapeutic approach for patients with moderate to severe AD (NCT: No. 00322153/Completed/Phase 3/Dementia of the Alzheimer's Type) [75, 76]. Consequently, gene editing therapies, targeting the underlying etiology, propose two effective strategies based on CRISPR/Cas9 technology: one involves correcting autosomal dominant mutations (e.g., in APP, PSEN1, or PSEN2) in induced pluripotent stem cell (iPSC) models to restore and stabilize the Aβ42/40 ratio [77]; the other entails disrupting pathogenic mutations in APP or inhibiting aberrant β‐secretase (β‐site APP cleaving enzyme 1)‐mediated cleavage of APP, thereby reducing toxic Aβ42 generation [78]. For SAD intervention, CRISPR/Cas9 can convert the APOE4 pathogenic allele to protective alleles APOE3 or APOE2, eliminating its role in promoting Aβ deposition and tau phosphorylation [79]; alternatively, it can reduce abnormal tau phosphorylation and NFT formation by knocking out the microtubule‐associated protein tau (MAPT) gene or editing its promoter region [80]. In delivery system research, Cas9–sgRNA complexes packaged into adenoviral vectors or nanoparticles have been delivered to neurons via intracerebral injection, offering potential strategies for targeted gene editing therapy [81, 82]. Furthermore, leveraging the targeted capabilities of CRISPR/Cas9, research teams have introduced or corrected AD‐associated gene mutations in human iPSC (hiPSC)‐derived brain organoids. This approach establishes isogenic cell lines, ultimately generating 3D brain organoids. These models recapitulate key AD pathological features, including Aβ accumulation, tau phosphorylation, and neuroinflammation, thereby providing a valuable platform for drug discovery and personalized clinical treatment [83].

#### 4.1.2 Parkinson's Disease

PD is a progressive neurodegenerative disorder common in older adults [84], pathology involving abnormal aggregation of α‐Syn (SNCA) [85] and reactive oxygen species (ROS)‐mediated damage to mitochondrial DNA and cellular components [86]. In gene editing‐based PD research, CRISPR/Cas9‐mediated targeting has shown promise in reducing SNCA expression. Kantor and colleagues [87] developed a novel lentiviral vector system carrying gRNA, dCas9 nuclease, and the catalytic domain of DNA methyltransferase (DNMT)3A, which specifically targets hypomethylated CpG islands in the intron 1 region of SNCA to suppress its transcription by remodeling chromatin structure. This optimized lentiviral system enables efficient delivery to neurons derived from hiPSCs with precise and stable expression. Additionally, mitochondrial dysfunction plays a critical role in PD pathogenesis [88, 89]. Defects in nuclear genes such as PRKN and PINK1 disrupt electron transport chain (ETC) complex function, leading to reduced ATP production and excessive ROS generation—processes that induce oxidative damage to lipids, proteins, and DNA. Concurrently, abnormalities in the PRKN/PINK1 pathway impair the clearance of damaged mitochondria, exacerbating cellular stress [90]. Targeting these mechanisms, gene editing strategies include: delivering wild‐type genes to dopaminergic neurons via adeno‐associated virus (AAV) to repair loss‐of‐function mutations [91, 92]; directly correcting pathogenic mutations in nuclear genes with CRISPR/Cas9 to restore mitochondrial homeostasis [88, 92]; or redesigning ETC‐related genes, integrating them into the nuclear genome, and directing their protein products to mitochondria via mitochondrial targeting signals to compensate for ETC deficits [88, 93]. Furthermore, CRISPR/Cas9‐based knockout or overexpression of pathological proteins enables the generation of PD‐relevant gene‐deficient animal models, which are essential for recapitulating clinical symptoms and advancing therapeutic research [94]. Similarly, the team led by Wulansari et al. [95] utilized CRISPR–Cas9 technology to introduce a PD‐associated DNAJC6 loss‐of‐function mutation into human embryonic stem cells. These genetically modified stem cells were then differentiated into human midbrain‐like organoids, which model the midbrain developmental environment of the human embryo, generating vulnerable midbrain dopaminergic neurons [95]. DNAJC6 mutations lead to defects in clathrin‐mediated endocytosis and vesicular trafficking of lysosomal enzymes, impairing the internalization of WNT ligands with their receptor complexes. Consequently, this reduces the expression of key transcription factors (LMX1A and EN1) crucial for midbrain development and promotes the accumulation of abnormal proteins, such as α‐Syn [96, 97]. Furthermore, phenotypic rescue experiments demonstrated that overexpressing DNAJC6 in damaged neurons reversed these pathological changes, strongly supporting the therapeutic necessity of targeting DNAJC6 [95]. Utilizing patient‐derived iPSCs to generate organoids enables the effective testing of various drugs for efficacy against specific genetic mutations. Currently, the majority of clinical PD management focuses on symptomatic treatment, broadly stratified into: neuroprotective strategies; treatment of motor symptoms (primarily employing levodopa therapy); treatment of nonmotor symptoms (also typically involving symptomatic management) (NCT: No. 00354133/Active, not recruiting/Phase 4/Parkinson's Disease) [98]. However, these approaches fail to effectively slow disease progression, offering primarily symptomatic relief. Therefore, based on current research, gene editing therapy emerges as one of the key therapeutic strategies holding genuine promise for etiological treatment of PD.

#### 4.1.3 Huntington's Disease

HD is a severe autosomal dominant neurodegenerative disorder [99], characterized by the presence of mutant huntingtin protein (mHTT) in inclusion bodies, caused by abnormal expansion of cytosine–adenine–guanine (CAG) trinucleotide repeat sequences within the HTT gene [100]. In healthy individuals, the HTT gene typically contains approximately 18 CAG repeats, whereas HD patients carry 40 or more such repeats [99]. This abnormal expansion leads to the excessive elongation of polyglutamine tracts, resulting in the production of toxic mHTT [101]. The toxic aggregation of mHTT induces a cascade of neurotoxic effects, including mitochondrial dysfunction and excitotoxicity [102], while gene editing technologies targeting the CAG locus in the HTT gene can reduce or eliminate mHTT expression, thereby slowing the progression of neurodegeneration [60]. Currently, treatment for HD is solely symptomatic. Tetrabenazine is the only approved medication specifically for managing HD motor symptoms. Psychiatric and cognitive symptoms of HD are managed symptomatically with antipsychotic medications and nonpharmacological psychotherapy. However, this symptomatic approach primarily alleviates clinical manifestations and does not offer a cure (NCT: No. 00219804/Completed/Phase 3/Huntington's Disease) [103]. Nevertheless, targeted gene editing at the nucleotide level offers the potential for a cure. Among gene editing tools, ZFPs lack nuclease activity and instead block HTT gene transcription through specific DNA binding [104]. Engineered ZFPs can selectively recognize mutant CAG repeats without affecting normal HTT protein function. TALENs, which rely on dual variable repeat monomers for nucleotide recognition, exhibit higher specificity and editing efficiency than ZNFs [105]. However, TALENs require a specific nucleotide at the target DNA terminus, potentially limiting the range of targetable sequences [99]. Currently, TALEN‐based research in HD therapy remains limited and warrants further investigation [106]. The CRISPR/Cas9 system directs Cas9 nuclease to specific DNA regions via gRNA, with cleavage dependent on a PAM—a sequence typically containing 2–5 conserved nucleotides [107]. In HD treatment, Cas9 can target both ends of the CAG repeat region in the HTT gene, inducing double‐strand DNA breaks that prompt cellular repair mechanisms to reduce CAG repeat length or silence the pathogenic gene, thereby decreasing mHTT expression [108, 109]. Furthermore, studies demonstrate that CRISPR/Cas9 can effectively correct the mutant HTT gene within 3D organoids differentiated from iPSCs reprogrammed from somatic cells derived from HD patients. Neurons differentiated from these corrected iPSCs exhibit partial reversal of phenotypic abnormalities, robustly demonstrating the therapeutic potential of CRISPR/Cas9 [110]. Despite these advances, gene editing technologies face limitations such as genotoxicity, off‐target effects, and inefficient delivery vectors [109, 111]. Balancing their therapeutic potential with safety remains a critical focus for precision medicine in HD.

#### 4.1.4 Infectious Neurological Diseases

Infectious neurological diseases are a category of disorders characterized by infections of the CNS, particularly the brain, caused by various microorganisms [112]. Viruses, as a critical pathogenic factor, often contribute to the high morbidity and mortality associated with these conditions [113]. Currently, clinical management of infectious neurological disorders primarily relies on two main therapeutic approaches: direct antipathogen therapy and immunomodulatory therapy. Although these therapies exhibit strong specificity against their respective pathogens, prolonged use has led to the increasing development of antimicrobial resistance in microorganisms, progressively diminishing therapeutic efficacy. Particularly concerning is the absence of curative treatments for prion diseases. These challenges collectively highlight the critical need for novel therapeutic strategies [114]. Gene editing technologies, particularly the CRISPR/Cas9 system, offer innovative strategies for targeted therapy of CNS infections. As a versatile endonuclease, Cas9 enables precise genomic targeting through the design of gRNAs. For example, HIV‐1 establishes latent infection by permanently integrating its genome into host cells, including those in the nervous system, a process that traditional antiviral therapies cannot fully reverse [115, 116]. The CRISPR/Cas9 system, guided by specific gRNAs, can target the long terminal repeat sequences of integrated HIV‐1 proviruses, promoting the excision and degradation of viral DNA [117]. Introducing Cas9–gRNA complexes into uninfected cells confers HIV‐1 resistance without inducing off‐target edits in the host genome [118]. However, viral escape mutants may arise [119], necessitating combinatorial therapeutic approaches to minimize resistance [120]. The human neurotropic polyomavirus JC (JCV) is the cause of progressive multifocal leukoencephalopathy, a fatal demyelinating disease associated with immune dysfunction [121]. During immunosuppression, latent JCV disseminates to the CNS via the bloodstream, infecting glial cells and undergoing extensive replication, which leads to cell lysis, demyelination, and neuronal damage [121]. Viral replication involves the accumulation of large T antigen (T‐Ag), which initiates viral DNA replication and regulates the transcriptional switch for late gene expression. Studies demonstrate that lentiviral delivery of Cas9 paired with gRNAs targeting the T‐Ag coding region specifically eliminates JCV, a strategy with potential for broader application to other viral diseases [122]. Despite these advances, gene editing faces critical challenges: off‐target effects remain a concern, and current delivery systems lack cell‐specific targeting and pose biosecurity risks. Addressing these issues—particularly optimizing delivery system targeting and reducing toxicity—is pivotal for advancing gene editing toward clinical translation [118, 123].

### 4.2 Applications and Strategies of RNA Editing Therapy for Nervous System Disorders

The key enzyme families involved in RNA editing are mainly the ADAR and APOBEC families. With advancements in RNA sequencing technologies, the potential roles of RNA editing in the nervous system have been increasingly revealed [124]. Unlike gene editing, RNA editing typically does not induce permanent changes to the genome, as the modifications it mediates are reversible [125]. The roles of both ADAR and APOBEC families in neurological diseases have been reported, among which ADAR is more extensively studied due to its distinct expression patterns. Additionally, CRISPR–Cas13—an RNA‐targeting editing tool functionally analogous to the CRISPR/Cas9 system—exhibits greater flexibility in targeting sequences than Cas9 [126].

#### 4.2.1 Alzheimer's Disease

RNA editing plays a key role in AD. A‐to‐I editing patterns differ across brain regions in humans, and ADAR‐mediated RNA editing dysregulation may represent a key pathological factor in AD pathogenesis [127]. For instance, a hallmark of AD is the misfolding of MAPT into paired helical filaments, whose abnormal aggregation leads to increased NFTs [128]. Studies have shown that RNA editing influences tau protein homeostasis through two mechanisms: ADAR activation promotes the production of abnormal proteins from 12→10 tau circRNA while reducing the translation efficiency of normal 12→7 tau circRNA. Mutations introduced by ADAR during translation may further contribute to NFT formation in the brain [129]. In the hippocampus of AD patients, RNA editing at the GluA2 Q/R site is significantly reduced. This editing deficit increases Ca^2^⁺ permeability of α‐amino‐3‐hydroxy‐5‐methyl‐4‐isoxazolepropionic acid receptor (AMPAR) channels, triggering calcium overload in neurons and excitotoxicity, which in turn leads to hippocampal neuron degeneration and cognitive impairment [130]. The association between abnormal ADAR levels, NFT formation, and neuronal damage highlights the complex role of RNA editing in AD pathology. Additionally, decreased A‐to‐I editing in the hippocampus and brain blood vessels of AD patients results in the accumulation of unedited Alu dsRNA, activating IRF and NF‐κB signaling pathways. This drives the expression of interferon‐stimulated genes and proinflammatory cytokines, initiating chronic neuroinflammation that closely links to key AD features like Aβ plaques [131].

#### 4.2.2 Parkinson's Disease

In PD research, pathological inclusions have been identified in neuronal nuclei, formed by the association of NONO/SFPQ proteins and A‐to‐I edited RNAs [132]. These inclusions further sequester key RNA molecules, leading to nuclear retention of RNAs—including transcripts encoding axon guidance, synaptic function, and mitochondrial proteins—which in turn reduces the expression of their corresponding proteins, leading to synaptic degeneration and neuronal death. Studies have shown that ADAR1 inhibition reverses nuclear RNA retention, restores the expression of axon‐ and synapse‐related proteins, and reduces NONO/SFPQ protein aggregation and neuronal death [132]. Another study highlights the critical roles of PINK1 and Parkin in mitochondrial autophagy within neurons [133]. PINK1 phosphorylates Parkin and mitochondrial‐associated proteins through its kinase activity, triggering Parkin's translocation from the cytoplasm to damaged mitochondria—a key step in initiating mitochondrial autophagy. However, the W437X mutation in the PINK1 gene impairs its ability to recruit Parkin, blocking mitochondrial autophagy and causing abnormal mitochondrial accumulation, which ultimately leads to neuronal damage [133]. By designing targeted gRNAs to mimic the R/G site structure of the native GluR2 transcript, ADAR2 can be directed to the W437K mutation site in the PINK1 gene, enabling gene repair. The restored PINK1 successfully recruits Parkin to damaged mitochondria, promoting mitochondrial autophagy and improving PD‐related pathological processes [134]. Additionally, ADAR1 deficiency activates proinflammatory responses in microglia and astrocytes of PD patients, worsening neuroinflammation and speeding up dopaminergic neuron damage [135].

#### 4.2.3 Amyotrophic Lateral Sclerosis

ALS is a motor neuron disease predominantly affecting adults, characterized by degeneration of motor neurons in the cortex, spinal cord, and brainstem. This leads to progressive muscle weakness, with patients often succumbing to respiratory failure [136]. Although causes of ALS have been identified, the precise mechanisms underlying motor neuron degeneration remain incompletely understood [137], and current therapeutic interventions for ALS have limited benefit [138]. Currently, clinical management of ALS emphasizes multidisciplinary comprehensive care aimed at improving patient quality of life, while therapies targeting disease modification have yet to demonstrate definitive efficacy. Riluzole, edaravone, and sodium phenylbutyrate/taurursodiol represent among the limited number of approved drugs that aim to mitigate cellular stress responses, thereby potentially slowing disease progression (NCT: No. 01492686 /Completed/Phase 3/Amyotrophic Lateral Sclerosis) [139, 140]. ALS is classified into rare familial cases (5–10%) and sporadic cases (>90%) [136]. A key pathogenic mechanism in sporadic ALS involves significantly reduced ADAR2 expression in motor neurons, leading to aberrant editing at the Q/R site of the GluA2 subunit of AMPARs. This abnormality converts AMPARs into calcium‐permeable channels, causing excessive Ca^2^⁺ influx and intracellular calcium overload, which in turn triggers cleavage of TAR DNA‐binding protein 43 (TDP‐43) [141, 142]. Targeting this pathway, AAV9 vectors—capable of crossing the blood–brain barrier—have been used to deliver the ADAR2 gene to ADAR2‐deficient motor neurons. This approach restores ADAR2 activity and halts the progression of ALS phenotypes [143]. Additionally, oral administration of perampanel has been shown to inhibit disease progression in individuals with ADAR2 dysfunction [144].

#### 4.2.4 Glioblastoma

Glioblastoma (GBM) is a highly invasive primary malignant brain tumor that occurs in both adults and children [145]. Its tumor tissue harbors stem‐like cancer cells (glioma stem cell [GSCs]), which contribute to therapeutic resistance and tumor recurrence [146]. Clinically, effective treatment modalities for GBM remain limited to approaches such as chemotherapy and surgical intervention. However, due to GBM's complex molecular characteristics and extensive heterogeneity, recurrence and mortality rates remain persistently high. This underscores the urgent need for targeted therapeutic agents specifically designed for GBM [147, 148]. GSCs typically express stem cell markers, form tumor spheres in serum‐free conditions, and induce tumor growth in vivo [149], making targeting of GBM cells or GSCs critical for treatment. Studies have shown that ADAR1 increases GM2A expression via 3′‐UTR (untranslated regions) editing [150]. As a key activator of ganglioside GM2 catabolism, GM2A collaborates with HEXA/HEXB proteins to promote GM2 degradation in lysosomes [151, 152]—a process essential for maintaining GSC stemness. ADAR1 expression is regulated by TYK2; inhibition of TYK2 downregulates ADAR1, reduces GM2A levels, disrupts lysosomal GM2 metabolism in GSCs, and consequently suppresses their proliferation and tumor‐initiating capacity. Targeting the TYK2/ADAR1/GM2A axis could impair GSC stemness and boost chemotherapy response [150]. Another study reveals that ADAR2 acts on the noncoding region of CDC14B pre‐mRNA to upregulate CDC14B mRNA and protein expression [153]. As a phosphatase, CDC14B stabilizes cell cycle checkpoint proteins p21 and p27 by degrading Skp2 (an E3 ubiquitin ligase), thereby inhibiting the G1/S phase transition and blocking tumor cell proliferation [154]. Reduced ADAR2 editing activity and CDC14B expression in GBM lead to elevated Skp2 levels, promoting abnormal cell proliferation [153]. Therefore, boosting ADAR2 activity in GBM could be an effective treatment strategy.

In summary, rapid progress in gene and RNA editing has opened new paths for precise treatment of nervous system disorders. Gene editing acts by directly correcting mutated genes or regulating key pathway genes, offering long‐lasting effects but inducing permanent genetic alterations; conversely, RNA editing dynamically modulates posttranscriptional modifications, avoiding long‐term genetic changes yet suffering from uncontrollable reaction dosages [155]. Studies have demonstrated that single‐gene lesions can trigger diverse neuronal damages, which may manifest as different neurological diseases across populations [156], highlighting the need for personalized precision targeted therapy. Central to this effort is the challenge of avoiding excessive editing and precisely controlling the maintenance or elimination of editing processes. The blood–brain barrier severely restricts the efficiency of intracerebral vector delivery, necessitating the development of novel nanoparticles or engineered viral vectors to enhance penetration. Despite these challenges, both editing technologies exhibit enormous potential superior to conventional treatments, making the acceleration of their translational applications a critical focus in nervous system disease research. Importantly, current editing techniques are fraught with potential off‐target effects and biosafety concerns. Future work must focus on better delivery systems, increased editing specificity, and long‐term efficacy studies to close the gap between research and clinic. Additionally, the importance of multidisciplinary combinatorial therapies cannot be overstated, as they may represent a necessary pathway toward curing nervous system disorders. Table 1 summarizes the different applications and strategies of gene editing and RNA editing in the treatment of various neurological diseases.

**TABLE 1:** Therapeutic applications of gene editing and RNA editing in neurological disorder.

Disease category	Medical terminology	Enzyme	Molecular operating principle	Technical implementation strategy	References
Gene‐editing enzyme	Frontotemporal dementia	Cas9	To address the pathogenic mutation in the CHMP2B gene within induced pluripotent stem cells (iPSCs) derived from frontotemporal dementia (FTD) patients, the research team employed CRISPR/Cas9‐mediated targeted gene correction, with single‐stranded oligodeoxynucleotides (ssODNs) as repair templates to precisely rectify the mutated locus. Following gene correction, the aberrant mRNA was eliminated, and normal mRNA splicing process. Ultimately, through the generation of isogenic control cell models with corrected CHMP2B, it was validated that pathological phenotypes induced by the CHMP2B mutation—including mitochondrial dysfunction, abnormal expansion of endosomal structures, and iron homeostasis imbalance—could be effectively reversed. These findings establish the direct pathogenic role of CHMP2B gene mutations in disease pathogenesis.	*Disease model construction*: The research team first generated iPSCs from skin fibroblasts of an FTD3 patient using nonintegrating plasmid reprogramming technology, a method that effectively minimizes the risk of exogenous gene integration and preserves genomic stability. Subsequently, CRISPR/Cas9‐mediated targeted genome editing was applied to patient‐derived iPSCs. By using the homology‐directed repair (HDR) mechanism, pathogenic mutations were precisely corrected, leading to the successful establishment of isogenic control cell lines with matched genetic backgrounds. *Neuronal differentiation and phenotypic analysis*: The research team directed patient‐derived iPSCs and isogenic control iPSCs with corrected CHMP2B mutations to differentiate into forebrain cortical neurons. Using morphological observation, ultrastructural analysis, functional assays, and transcriptomic sequencing, they systematically compared the phenotypic differences between mutant and gene‐corrected cell lines.	[157]
Gene‐editing enzyme	Duchenne muscular dystrophy	Cas9	The research team utilized single‐guide RNAs (sgRNAs) to direct Cas9 nuclease for double‐strand DNA cleavage at specific loci of the target gene. By precisely disrupting splice donor or acceptor sites within the target exons, they induced selective skipping of these exons during transcription, thereby restoring the correct open reading frame (ORF) of downstream exons. Targeting the splice acceptor sites of exons 53 and 44 in the dystrophin‐encoding gene, the team engineered exon skipping and reading frame correction via a frameshift, ultimately restoring functional dystrophin expression.	*Single‐nick editing strategy*: The single‐nick gene editing strategy developed by the research team requires only designing an sgRNA to guide Cas9 nuclease for precise cleavage at target genomic loci. This strategy integrates the advantages of high editing efficiency and minimal genomic alterations, enabling the design of mutation‐specific sgRNAs for diverse pathogenic variants. As such, it establishes a flexible and safe technical framework for the precise implementation of gene therapy. *Delivery system design*: The research used an ssAAV9 vector to deliver Cas9 nuclease and an scAAV9 vector to carry sgRNA. Via these two vectors, the efficiency of delivering gene‐editing components, Cas9 and sgRNA, was significantly improved. *Cross‐species validation*: The research team validated the efficacy of sgRNAs in human iPSC‐derived cardiomyocytes, confirming the clinical potential of the gene‐editing system.	[158]
Gene‐editing enzyme	Dravet syndrome	dCas9	Dravet syndrome is primarily caused by heterozygous loss‐of‐function mutations in the human SCN1A gene, leading to a significant reduction in the expression of voltage‐gated sodium channel protein Na<sub>v</sub>1.1. The research team developed a dCas9–VP160 gene activation system by fusing catalytically inactive dCas9 with the superpotent transcriptional activation domain VP160. By designing mutation‐specific sgRNAs targeting the proximal promoter region of the mouse orthologous Scn1a gene, the dCas9–VP160 complex was directed to bind target regulatory elements. This recruitment of RNA polymerase enhanced Scn1a transcription efficiency, thereby restoring the electrical excitability of inhibitory interneurons and effectively alleviating the epileptic phenotype in animal models.	*Ex vivo screening and validation*: By bioinformatics analysis to interrogate the Scn1a promoter region, the team designed and tested multiple sgRNA libraries, ultimately identifying lead candidates with highly efficient targeting of the proximal promoter. The efficacy the dCas9–VP160 activation system was validated across P19 cells, primary neurons, and mouse embryonic fibroblasts (MEFs), demonstrating significant upregulation of Scn1a mRNA and Na_v1.1 protein expression. *In vivo delivery and therapy*: A dual AAV9 vector system was employed to package dCas9–VP160 and sgRNA components, achieving broad transduction in the brain via intracerebroventricular injection in neonatal mice. The system was designed with the Dlx5/6 enhancer driving rtTA expression, enabling selective activation in GABAergic interneurons while minimizing off‐target effects on excitatory neurons. *Functional restoration and phenotypic improvement*: In inhibitory interneurons of Dravet syndrome model mice, the dCas9–VP160 activation system restored action potential firing frequency and threshold, correcting excitability deficits. Following treatment, heat‐induced seizure thresholds in mice were significantly elevated, and seizure duration was notably reduced.	[159]
Gene‐editing enzyme	Epilepsy	dCas9	Mechanisms of epilepsy treatment via CRISPR activation technology: The research team utilized CRISPR activation (CRISPRa) to target the promoter region of the potassium channel gene Kcna1, leading to specific upregulation of its transcriptional activity. The voltage‐gated potassium channel protein encoded by this gene enhances the repolarization of neuronal membrane potentials, effectively decreasing action potential firing frequency and suppressing abnormal synchronous discharge in neuronal networks. These effects collectively reduce the frequency and intensity of spontaneous tonic–clonic seizures.	*Target gene selection*: Key genes involved in regulating neuronal excitability were identified through bioinformatic analyses, with their promoter regions characterized to facilitate the design of specific sgRNAs. *Delivery system*: A dual AAV9 vector system was employed to deliver the transcriptional activator dCas9–VP64 and sgRNAs, ensuring efficient, neuron‐specific transduction. The Camk2a promoter was utilized to restrict gene activation to excitatory neurons, thereby minimizing off‐target effects. *Inducible control system*: A doxycycline‐inducible system was employed to activate the CRISPR machinery only when doxycycline is present, enabling precise control over gene expression regulation.	[160]
Gene‐editing enzyme	Charcot‐Marie‐Tooth	Cas9	Charcot‐Marie‐Tooth disease is caused by PMP22 gene duplication mutations, which lead to overexpression of the PMP22 protein and subsequent myelin loss in peripheral nerves. The research team used a CRISPR/Cas9 genome‐editing system to target and disrupt the TATA‐box core regulatory region within the P1 promoter of the PMP22 gene. This intervention specifically lowered PMP22 transcriptional levels, effectively suppressing excessive protein expression. Experimental results demonstrated that this approach alleviated demyelinating lesions, increased myelin sheath thickness, and reduced the proportion of “onion bulb” structures and unmyelinated axons, thereby improving the pathological phenotype of peripheral nerves at the molecular level.	*Nonviral delivery system*: Preassembled ribonucleoprotein (RNP) complexes were used to circumvent the potential immunogenicity of viral vectors, offering a safer alternative for delivery of gene‐editing components. *Intervention timing*: Preventive treatment via injection prior to disease onset significantly reduced demyelination and axonal degeneration, whereas therapeutic intervention with post‐onset injection partially improved pathological indices. These findings demonstrate the treatment's potential to mitigate disease progression across different intervention windows.	[161]
Gene‐editing enzyme	Schizophrenia	Cas9, TALEN	They used TALEN and CRISPR/Cas9 gene‐editing technologies to precisely repair pathogenic mutations in the schizophrenia‐associated DISC1 gene, effectively restoring structural and functional deficits in neuronal synapses. Targeted editing of key neurodevelopmental genes—KCTD13 and FGFR1—was performed to investigate their regulatory roles in signaling pathways, including Wnt/β‐catenin, RhoA–ROCK, and NF‐κB. These investigations clarified the molecular mechanisms by which these genes govern critical neurodevelopmental processes, such as neural cell differentiation, axonal guidance, and synaptic plasticity.	*Construction of genetically engineered cell models*: These models were developed to rapidly validate the effects of candidate genes on synaptic protein expression and neuronal differentiation. Additionally, they enable the investigation of schizophrenia‐associated gene functions in non‐neuronal cells and the development of drug screening platforms for therapeutic discovery. *Complex multicellular models*: Key genes were edited to model cortical developmental abnormalities, enabling investigations into the molecular mechanisms governing cell proliferation, laminar organization, and excitatory/inhibitory (E/I) balance. By integrating neurons and glial cells, these models facilitated studies on the pathological roles of synaptic pruning and inflammatory factors in schizophrenia, shedding light on their contributions to disease pathogenesis. *Drug development*: Gene‐edited cell models were utilized to assess the capacity of candidate drugs to reverse abnormal phenotypes, providing a preclinical platform for therapeutic efficacy evaluation.	[162]
Gene‐editing enzyme	GM2 gangliosidosis	Cas9	This disorder arises from mutations in the lysosomal enzyme‐encoding genes HEXA or HEXB, leading to the abnormal accumulation of the substrate GM2 ganglioside in lysosomes due to impaired degradation. Using CRISPR–Cas9 gene‐editing technology, the research team introduced a synthetic Hex μ‐subunit‐encoding gene (HEMX) into the liver genome, enabling stable expression of HEMX in liver cells. Secreted into the bloodstream, HEMX protein traverses the blood–brain barrier via systemic circulation and forms catalytically active homodimers in the central nervous system. These homodimers mimic the collaborative function of native HEXA and HEXB subunits, effectively degrading the abnormally accumulated GM2 ganglioside and reversing lysosomal storage pathology at the molecular level.	*Targeted safe harbor integration*: The CRISPR–Cas9 system was used to precisely integrate a promoter‐deleted HEXM cDNA into the mouse albumin locus, leveraging the endogenous albumin promoter to drive constitutive HEXM expression. This approach mitigates risks associated with random genomic integration by utilizing a well‐characterized safe harbor locus, ensuring stable and tissue‐specific transgene expression. *Dual AAV8 vector system design*: The AAV8–SaCas9 vector carries Staphylococcus aureus Cas9 (SpCas9), driven by a liver‐specific promoter to restrict gene editing activity exclusively to hepatocytes. Concurrently, the AAV8–HEXM–sgRNA vector contains a single‐guide RNA (sgRNA) targeting the albumin genomic locus and the HEXM donor sequence, enabling precise homology‐directed repair at the predetermined safe harbor site. *Pathological improvement*: Reduced cellular vacuolization was observed in hepatic and neuronal tissues, with GM2 ganglioside levels normalized in the liver, heart, and spleen.	[163]
Gene‐editing enzyme	Fragile X syndrome	Cas9	The disorder arises from the abnormal expansion of a CGG trinucleotide repeat sequence within the 5′UTR of the FMR1 gene, triggering DNA hypermethylation and chromatin conformational remodeling in the gene promoter region. These epigenetic alterations ultimately lead to silencing of FMR1 gene expression. The research team designed sgRNAs targeting both ends of the CGG repeat tract, directing Cas9 nuclease to induce double‐strand DNA breaks at the termini of the expanded repeats. During the DNA repair process, the nonhomologous end joining (NHEJ) mechanism mediated precise or near‐precise excision of the CGG repeats, generating FMR1 functional alleles devoid of repeat expansions. This genetic structural correction eliminated the triggering signals for DNA hypermethylation and chromatin remodeling, significantly reducing methylation levels in the FMR1 promoter and restoring transcriptional activity. The reactivated FMR1 gene produces functional FMRP protein through transcription and translation, thereby restoring its critical regulatory functions in neurodevelopment.	*Dual sgRNA design*: Two single‐guide RNAs (sgRNAs) were designed to target the upstream and downstream flanking regions of the CGG repeat tract, ensuring complete excision of the expanded repeats while preserving the gene's transcription start site (TSS). *Patient‐derived iPSC cell model*: IPSCs derived from patient samples were utilized to validate the feasibility of CRISPR‐mediated genome editing in human pluripotent stem cells, modeling the therapeutic potential for clinical applications. *Limitations and optimization*: Cell division rate impacts epigenetic reprogramming: rapidly dividing somatic cells are more prone to methylation loss, while iPSCs exhibit relatively low CRISPR activation efficiency.	[164]
Gene‐editing enzyme	Spinal muscular atrophy	Cas9	This disorder is primarily caused by the deletion of the survival of motor neuron 1 (SMN1) gene, leading to insufficient functional SMN protein. The paralogous gene SMN2 cannot effectively compensate for SMN1 loss due to an abnormal splice acceptor site in exon 7. Conserved splicing silencer elements—such as CUG repeat sequences—within intron 7 of SMN2 recruit splicing repressor proteins like hnRNPA1/A2, hindering exon 7 inclusion and reducing the production of functional full‐length SMN protein. Using the CRISPR/Cas9 gene‐editing system, the research team achieved targeted disruption of these intronic splicing silencer elements in SMN2, alleviating splicing repression. This intervention significantly promoted the correct splicing and inclusion of exon 7, thereby increasing functional full‐length SMN protein.	*In vitro model*: sgRNAs were designed to target the ISS‐N1 and ISS+100 sites within the SMN2 intron. Following transfection into iPSCs derived from SMA patients, Cas9‐mediated double‐strand breaks were induced, disrupting splicing regulatory elements (SREs) via NHEJ. In splice‐corrected iPSCs, the inclusion rate of exon 7 was significantly elevated, accompanied by restored motor neuron survival and reduced apoptosis. *Systemic restoration in mouse models*: Genome‐edited mice exhibited substantial increases in full‐length SMN (SMN‐FL) protein levels across multiple tissues—including the spinal cord and brain—alongside improved motor function. Quantitative analyses revealed recovery of neuromuscular junctions and motor neuron counts, reflecting structural and functional restoration of the neuromuscular system. *Durability*: Genomic editing achieved permanent splice correction, offering a mechanistic advantage over conventional therapies that require repeated administration. This approach eliminates the need for continuous intervention by integrating heritable genetic modifications, establishing a durable solution for long‐term disease management.	[165]
Gene‐editing enzyme	Rett syndrome	Cas9	This neurodevelopmental disorder is caused by mutations in the methyl‐CpG binding protein 2 (MeCP2) gene located on the X chromosome. The research team designed gRNAs targeting pathogenic mutation sites to direct Cas9 nuclease for specific double‐strand breaks within the mutated MeCP2 gene. By leveraging a donor DNA template containing wild‐type sequences, precise correction of the mutation was achieved via the HDR mechanism. This intervention successfully restored normal expression levels of the MeCP2 protein, addressing the molecular deficit underlying Rett syndrome.	*Magnetic nanoparticle delivery system*: Cationic polymers, CRISPR–Cas9 nucleases, and donor DNA were electrostatically loaded onto the surface of magnetic nanoparticles, enabling codelivery of multiple plasmids in a single transfection event. This modular design leverages electrostatic interactions to stabilize nucleic acid‐polymer complexes, ensuring efficient cargo encapsulation and cellular uptake. *Magnetofection technology*: External magnetic fields were applied to accelerate nanoparticle‐cell binding, enhancing transfection efficiency to 99.3% while reducing incubation time by 40% compared with conventional methods. The magnetic guidance system promotes rapid accumulation of nanoparticles at the cell membrane, overcoming diffusion limitations and optimizing reagent utilization. *Magnetic activated cell sorting*: Magnetic selection was employed to enrich cells that successfully internalized the nanoparticles, achieving a repair efficiency of 42.95%. This label‐dependent sorting strategy selectively isolates cells with high nanoparticle uptake, minimizing contamination from untransfected populations and improving the purity of genetically modified cell pools.	[166]
Gene‐editing enzyme	Friedreich ataxia	Cas9	The disorder arises from the abnormal expansion of a GAA trinucleotide repeat within intron 1 of the FXN gene. This aberrant repeat induces the formation of repressive chromatin states, leading to silencing of FXN expression. To address this molecular defect, CRISPR/Cas9‐based approaches employ two distinct strategies: targeted repeat excision, sgRNAs are designed to recognize the 5′ and 3′ boundaries of the expanded GAA tract, enabling precise excision of the pathological repeats; intron 1 deletion, alternatively, sgRNAs target the upstream and downstream boundaries of FXN intron 1, facilitating complete removal of the entire intron while preserving critical exon splicing regulatory regions. The latter strategy achieves more complete elimination of repressive chromatin structures, potently restoring FXN transcriptional activity. Functional validation demonstrates that this intervention significantly increases the number and size of axonal mitochondria and restores the structural integrity of synapses, reversing FRDA‐associated pathological phenotypes at both molecular and cellular levels.	*Design of gene editing strategies*: Targeting regions immediately flanking the GAA repeat tract to excise only the expanded repeats while sparing adjacent genomic sequences; targeting the distal boundaries of intron 1 to delete large intronic regions containing the pathological repeats, ensuring preservation of critical exon splicing signals to maintain proper pre‐mRNA processing. *Cellular and structural outcomes*: This intervention restored neuronal survival, axonal morphology, and synaptic formation capacity in dorsal root ganglion organoids, reflecting functional recovery at both cellular and subcellular levels.	[167]
Gene‐editing enzyme	Diffuse midline glioma	Cas9	Specific guide RNAs (gRNAs) were designed to target tumor‐suppressor genes (Trp53, Pten, Atm, Cdkn2a). The CRISPR/Cas9 system was then employed to precisely knock out these genes, mimicking the characteristic gene mutation profiles of human DMG. The research team codelivered the gRNAs and the oncogene PDGF‐B into Nestin⁺ neural stem cells expressing the TVA receptor. Through receptor‐mediated targeted delivery on the cell surface, cell‐type‐specific gene editing was achieved. Meanwhile, the Cre/LoxP recombinase system was used to activate the expression of the Cas9 gene at the Rosa26 locus. Through spatio‐temporal specific regulation, gene editing events were ensured to occur only in the target neural stem cells, avoiding off‐target effects and enhancing the tissue‐targeting ability of the editing system.	*Generation of genetically engineered mice*: NNC genotype mice (Nestin–tv‐a; Nestin–Cre; Rosa26‐LoxP–Stop–LoxP–Cas9–EGFP) were generated through strategic crossings, enabling cell‐type‐specific expression of Cas9 in neural stem cells. *Viral delivery*: The RCAS virus carrying gRNA and PDGF‐B was injected into the midline of the brains of neonatal mice. This allowed the virus to infect tv‐a⁺ neural stem cells and initiate gene editing. *Multiplex gRNA pool design*: gRNAs targeting distinct genes (Trp53, Pten, Atm, Cdkn2a) were combined with control gRNAs to form a pooled library. This gRNA cocktail was codelivered via the RCAS virus, enabling simultaneous perturbation of multiple tumor‐suppressor pathways to mimic the complex genetic interaction landscape of human DMG.	[168]
Gene‐editing enzyme	Ependymoma	Cas9	The research team designed sgRNAs targeting the breakpoint regions of C11orf95 and RELA on human chromosome 11. Using the CRISPR/Cas9 system, they induced double‐strand DNA breaks at these specific genomic loci. Activation of the NHEJ repair pathway promoted aberrant ligation of the broken gene ends, culminating in the formation of the oncogenic C11orf95–RELA fusion gene. This strategy faithfully recapitulates the chromothripsis‐driven gene rearrangements characteristic of human ependymoma, establishing a pivotal model for dissecting the molecular mechanisms tumor initiation and progression.	*Targeted gene editing design*: Multiple pairs of sgRNAs were designed targeting specific exons of the human C11orf95 and RELA genes to mimic the breakpoints caused by chromothripsis. *Functional validation of oncogenicity of fusion genes*: In vitro experiments confirmed the expression of CRISPR/Cas9‐induced fusion genes in human 293T cells and mouse NIH3T3 cells. Fusion transcripts and protein products were characterized using RT‐PCR, Sanger sequencing, and Western blot analysis, providing molecular evidence of successful gene rearrangement; in vivo experiments involved delivering sgRNAs and Cas9 into mouse neural stem cells in the brain via lentiviral vectors. This approach induced formation of endogenous RELA fusion genes, driving the development of ependymoma‐like tumors. These experiments recapitulated the oncogenic potential of chromothripsis‐derived gene fusions, establishing a causative link between genomic rearrangements and tumorigenesis in a mammalian model. *In vivo tumor model construction*: The pTomo lentiviral vector was employed to deliver sgRNAs and Cas9, allowing cell‐specific gene editing by targeting Nestin⁺ neural stem cells via intracranial injection. This approach harnessed the tropism of lentiviral vectors to preferentially transduce neural stem cells, enabling precise genomic manipulation within the central nervous system. By combining tissue‐specific delivery with CRISPR/Cas9‐mediated gene editing, the strategy ensures targeted disruption of oncogenic pathways in Nestin‐expressing neural progenitors, a critical cell population implicated in ependymoma initiation.	[169]
Gene‐editing enzyme	Angelman syndrome	Cas9	In neurons, the paternal UBE3A (patUBE3A) allele undergoes transcriptional silencing mediated by the long noncoding RNA UBE3A–ATS. The Snord115 gene cluster—encoding a small nucleolar RNA (snoRNA) cluster located in the 3' region of UBE3A–ATS—exhibits expression levels closely associated with the neuron‐specific transcriptional activity of UBE3A–ATS. The research team utilized the CRISPR/Cas9 system to design gRNAs targeting multiple tandem repeat sequences within the Snord115 gene cluster. By inducing double‐strand breaks at specific genomic loci, this approach disrupted the transcriptional elongation of UBE3A–ATS, thereby specifically relieving transcriptional silencing of the patUBE3A allele.	*Target selection and gRNA design*: Through systematic screening, we identified a highly efficient gRNA targeting SNORD115. This gRNA is capable of simultaneously binding to approximately 75 SNORD115 repeat sequences, thereby maximizing transcriptional interference efficacy. *Optimization of the delivery system*: To accommodate the limited packaging capacity of AAV vectors, the smaller SaCas9 was employed as a substitute for SpCas9. The AAV9 serotype was utilized to deliver SaCas9 and gRNA, leveraging its high efficiency in crossing the blood–brain barrier (BBB) to achieve neuron‐specific expression. *Phenotypic rescue validation*: The intervention restored brain weight, alleviated microcephaly, and improved motor coordination while reducing stereotyped behaviors. Notably, no neuroinflammation, off‐target effects, or tumor formation were detected.	[170]
RNA‐editing enzyme	Rett syndrome	ADAR2	RNA editing technology was used to correct a guanine (G) to adenine (A) mutation in Mecp2 mRNA, disrupting the coding sequence. This intervention enabled the translated methyl‐CpG binding protein 2 (MECP2) to regain its biological function of specifically binding to methylated DNA, thereby reversing the pathological phenotypes of neural cells.	*Engineered editing enzyme design*: The catalytic domain of ADAR2 was fused with a bacteriophage‐derived RNA‐binding domain to create a hybrid protein. This RNA‐binding domain specifically recognizes and binds to the BoxB stem‐loop structure within the guide RNA, ensuring precise targeting of the editing enzyme to the desired mRNA. Additionally, a nuclear localization signal (NLS) was incorporated to direct the fusion protein to the nucleus, enabling direct action on newly transcribed Mecp2 pre‐mRNA. *Design and optimization of guide RNA*: The guide RNA was engineered to contain a sequence complementary to the mutated region of the target mRNA, with a strategically introduced C mismatch at the target adenosine (A) site. Additionally, two BoxB stem‐loop structures were incorporated flanking the target A site at both the 5' and 3' ends.	[171]
				*Viral delivery system*: Recombinant AAV vectors were employed to deliver the engineered editing enzyme and guide RNA to neuronal cells. The AAV construct incorporates a neuron‐specific promoter to ensure efficient, cell‐type‐restricted expression of the editing system.	
RNA‐editing enzyme	Epilepsy	ADAR2	RNA editing modifies the Q/R editing sites of glutamate receptor subunits (GLUR2, GLUR5, GLUR6), precisely regulating the ion channel properties of these receptors. In the temporal cortex tissue of epilepsy patients, RNA editing efficiency for GLUR5 and GLUR6 subunits is significantly increased, a phenomenon that may represent an adaptive regulatory response by which the body reduces Ca^2^⁺ influx to suppress abnormal epileptic activity.	*Targeted RNA editing enzymes*: Through precise modulation of ADAR (adenosine deaminase acting on RNA) enzyme activity, we can selectively enhance or suppress the editing of specific receptor subunits. This targeted approach enables fine‐tuning of Ca^2^⁺ influx and neuronal excitability, offering a potential therapeutic strategy for neurological disorders associated with excitability dysregulation. *Single‐cell level investigation*: By integrating single‐cell PCR technology, we analyzed the RNA editing profiles within specific neuronal populations. This approach revealed significant editing heterogeneity among individual neurons, providing novel insights into its potential contribution to epileptogenesis. *Electrophysiological validation and drug development*: Electrophysiological characterization was performed to validate functional alterations in edited receptor variants. Based on these findings, we conducted high‐throughput screening to identify compounds that mimic the pharmacological properties of the R‐form receptors, thereby facilitating the development new antiepileptic therapeutics.	[172]
RNA‐editing enzyme	Acute spinal cord injury	ADAR2	After acute spinal cord injury, the RNA editing level at the R/G site of AMPA receptor subunits (GluR2, GluR3, GluR4) significantly decreases. The reduction in the editing level at this site slows down the resensitization kinetics of the AMPA receptor ion channels. This, in turn, weakens the responsiveness of postsynaptic neurons to the neurotransmitter glutamate, subsequently reducing Ca^2^⁺ influx and alleviating excitotoxicity damage. Ultimately, this limits neuronal death during the acute phase of the injury.	*Targeted modulation of ADAR2 activity*: They developed pharmacological and gene therapy approaches to enhance ADAR2 enzymatic activity, aiming to restore physiological editing levels at R/G sites. This strategy achieves a dual therapeutic objective by balancing neuroprotective effects during the acute phase with the restoration of synaptic plasticity requirements during the recovery phase. *Regulation of AMPA receptor function*: Design of selective AMPA receptor antagonists aims to mitigate excessive excitotoxicity during the acute phase of injury while preserving long‐term synaptic plasticity. These compounds specifically target overactive AMPA receptor‐mediated calcium influx, a key driver of excitotoxic neuronal death, without compromising the synaptic remodeling essential for functional recovery. By balancing acute neuroprotection and chronic synaptic integrity, this strategy addresses a critical therapeutic window in neurological disorders characterized by excitatory dysfunction, such as spinal cord injury and stroke. *Combined interventions*: Integrating anti‐inflammatory therapy—such as inhibiting inflammation‐associated upregulation of ADAR1—and RNA editing regulation aims to mitigate secondary injury and promote neurorepair. This synergistic approach targets dual pathological pathways: suppressing excessive inflammation‐driven ADAR1 activation, which exacerbates RNA misediting and cellular stress, while enhancing beneficial RNA editing events that facilitate axonal regeneration and synaptic remodeling. By addressing both the inflammatory cascade and molecular dysregulation, this strategy offers a comprehensive solution to combat the multifactorial nature of neurological damage, particularly in conditions like spinal cord injury and stroke where secondary tissue degeneration profoundly impacts outcomes.	[173]
RNA‐editing enzyme	Multiple sclerosis	APOBEC‐1	In the 3′UTR of the B2m gene, APOBEC1‐mediated cytosine‐to‐uracil (C‐to‐U) RNA editing may alter microRNA binding sites, thereby influencing the expression of β2‐microglobulin (B2m). As a core component of MHC‐I molecules, aberrant B2m expression is closely associated with the activation of proinflammatory responses and the progression of neurodegeneration. Additionally, APOBEC1‐deficient mice exhibit more severe neuroinflammatory phenotypes in the EAE model, shown by significantly increased microglial density, abnormal astrocyte proliferation, and enhanced T lymphocyte infiltration in spinal cord tissue.	*Functional validation using gene knockout models*: APOBEC1 knockout mice were generated and subjected to EAE induction. Clinical scores, inflammatory infiltration area, and degree of demyelination were compared between knockout and wild‐type mice to define the protective role of APOBEC1 in neuroinflammation. *Targeted pathway intervention*: Leveraging results from enrichment analysis, this study investigates how APOBEC1‐edited target genes modulate inflammatory responses, thereby identifying potential targets for developing RNA editing‐based therapeutic strategies.	[174]
RNA‐editing enzyme	Duchenne muscular dystrophy	ADAR2	Approximately 10% of muscular dystrophy patients harbor nonsense point mutations that generate premature termination codons (PTCs), leading to premature truncation of dystrophin translation. The mini‐dCas13X‐mediated RNA adenine base editor (mxABE), built from the compact dCas13X protein and the ADAR2 deaminase domain, targets specific RNA sequences to precisely convert A in termination codons to G, thereby converting them into nontermination codons. This editing process enables mRNA to bypass aberrant termination signals, guiding ribosomes to continue translating the full open reading frame and restoring the expression of functional dystrophin to rescue muscle function. Leveraging the compact nature of dCas13X, the mxABE system achieves precise localization and base editing of disease‐causing RNA sequences, presenting an innovative strategy for gene therapy of nonsense mutation‐related disorders.	*In vitro screening and optimization*: Efficient gRNAs were screened using a dual‐fluorescent reporter system to identify sequences with optimal targeting efficiency. *In vivo delivery system*: The mxABE editor was packaged into AAV9 vectors for local or systemic delivery, enabling efficient targeting of therapeutic RNA sequences in vivo. *Safety and specificity*: Bystander editing of adjacent adenines was detected in targeted RNA sequences, yet no new premature termination codons were introduced. Transcriptome analysis via RNA sequencing revealed no significant off‐target effects, demonstrating the high specificity of the mxABE system.	[175]
RNA‐editing enzyme	Schizophrenia	ADAR2, ADAR3	RNA editing levels are significantly decreased in the brains of individuals with schizophrenia (SCZ). Further analysis reveals that aberrant RNA editing sites within the 3′UTR of brain‐expressed genes substantially affect the expression patterns of mitochondrial‐related genes, potentially disrupting mitochondrial functional homeostasis through posttranscriptional regulatory pathways. Functional characterization of the MFN1 gene—critical for mitochondrial dynamics—shows that editing levels at two key sites are notably reduced in SCZ patients. Mechanistic experiments demonstrate that these editing site alterations directly impair mitochondrial fusion capacity and induce increased apoptosis, phenomena closely associated with abnormal mitochondrial dynamics and neurodegenerative changes observed in SCZ pathophysiology.	*Regulation of targeted editing enzymes*: Inhibiting ADAR3 activity and restoring normal ADAR2 function, to improve RNA editing levels. *Intervention at specific editing sites*: ASOs were designed to target editing sites within the 3'UTR, aiming to modulate the mRNA stability or translational efficiency of mitochondria‐related genes. This targeted approach leverages ASOs to specifically bind editing‐prone sequences in the 3′UTR, thereby influencing posttranscriptional regulatory mechanisms that govern gene expression. By fine‐tuning RNA editing at these sites, the strategy seeks to restore normal expression profiles of mitochondria‐related genes, potentially addressing dysfunctions in mitochondrial bioenergetics and dynamics associated with various pathological conditions. *Personalized medicine and consideration of ethnic disparities*: Studies reveal that individuals with SCZ of African ancestry exhibit elevated RNA editing levels, contrasting with trends observed in European patients. Future therapeutic strategies must incorporate genetic background differences to develop ethnic‐specific interventions, addressing the distinct molecular profiles underlying SCZ across populations.	[176]

## 5 Applications of Gene Editing and RNA Editing in the Treatment of Immune System Diseases

Immune system diseases, such as immunodeficiency disorders and autoimmune diseases, have complex etiologies involving genetic and environmental factors, epigenetic regulation, and immune cell dysfunction [177, 178]. Conventional therapies, including hormone therapy, immunosuppressants, targeted therapy, and hematopoietic stem cell (HSC) transplantation, often face challenges such as limited efficacy, significant side effects, or donor matching difficulties [179, 180]. Although these treatments can alleviate symptoms, they frequently fail to address the root causes, severely impacting patients' quality of life and health while imposing substantial medical and economic burdens [181, 182]. In recent years, the rapid development of gene editing and RNA technologies has brought new hope for treating immune system diseases. Gene editing, with its efficiency and precision, holds great potential for correcting defects in immune‐related genes and modulating immune cell functions [183].

### 5.1 Applications and Strategies of Gene Editing in Immune System Diseases

Gene editing technologies, particularly the CRISPR/Cas system, have been applied to treat immune system diseases by repairing gene mutations or suppressing pathogenic genes, thereby reversing immunodeficiency or ameliorating immune dysregulation [184, 185]. The development of novel delivery systems, such as ENVLPE virus‐like particles, has further improved the delivery efficiency and safety of gene editing tools [186]. By targeting pathogenic genes or regulating immune pathways, gene editing holds promise for correcting immune system abnormalities, enabling long‐term remission or even cure [187, 188]. However, the clinical application of this emerging technology still faces challenges, including off‐target effects, optimization of delivery systems, and ethical concerns [188]. Therefore, in‐depth research into the applications and strategies of gene editing in immune system diseases carries significant scientific importance and will provide tangible clinical benefits for patients.

#### 5.1.1 Severe Combined Immunodeficiency

SCID includes a group of genetic disorders with over 20 identified associated gene mutations. It is characterized by severe impairment of T lymphocyte differentiation, often accompanied by functional or quantitative abnormalities in B lymphocytes and natural killer (NK) cells, leading to combined defects in cellular and humoral immunity [189, 190]. The disease course is characterized by severe and rapidly progressive symptoms, primarily including: severe, recurrent, and persistent infections; failure to thrive; and graft‐versus‐host disease (GVHD) engrafted by transplacental maternal T lymphocytes [191]. Novel gene editing technologies, such as CRISPR/Cas9, ZFNs, and TALENs, enable precise gene insertion or mutation correction at safe genomic loci. In a study on IL7R‐SCID, CRISPR/Cas9 technology combined with AAV6 transduction was used to introduce corrected IL7RA cDNA into T lymphocytes and HSCs, correcting IL7RA mutations, thus restoring T cell development and signaling [192]. Researchers also employed CRISPR/Cas9 to repair the RAG2 gene in CD34+ hematopoietic stem and progenitor cells (HSPCs) derived from RAG2‐SCID patients, successfully generating CD3+ T cells with diverse T cell receptor (TCR) repertoires [193]. Similarly, ex vivo lentiviral gene therapy of HSPCs for adenosine deaminase‐deficient SCID (ADA‐SCID) achieved high overall and event‐free survival rates, sustained ADA expression, metabolic correction, and functional immune reconstitution [194], demonstrating the feasibility of gene editing for SCID. ZFNs and TALENs have also been used for gene correction in SCID‐X1, but CRISPR has become mainstream due to its higher efficiency and ease of use [195]. In terms of vectors, early γ‐retroviral vectors posed leukemia risks due to insertional mutagenesis, prompting a shift toward safer lentiviral vectors. Optimized lentiviral designs reduce genotoxicity and have achieved stable T cell, B cell, and NK cell reconstitution in clinical trials without tumorigenesis [196]. Several studies have explored AAV vectors for gene delivery, though their limited cargo capacity restricts their use primarily to ex vivo editing [197].

#### 5.1.2 Chronic Granulomatous Disease

Chronic granulomatous disease (CGD) is a primary immunodeficiency caused by defects in the phagocyte NADPH (nicotinamide adenine dinucleotide phosphate) oxidase complex, leading to impaired ROS production, recurrent bacterial/fungal infections, and chronic inflammation [198]. CGD primarily manifests as recurrent and severe infections, granuloma formation (e.g., granulomatous colitis), and noninfectious inflammatory complications [199]. Five key genes encoding NADPH oxidase subunits have been identified [200, 201]. CRISPR/Cas9 corrects CYBB mutations in X‐linked CGD using NHEJ or HDR, restoring NADPH oxidase function in CD34+ HSCs and iPSCs [202]. ZFN technology has been used to insert functional CYBB into the AAVS1 safe harbor locus, minimizing insertional mutagenesis risks and demonstrating higher‐than‐normal gp91phox‐positive cells in mouse models [203]. Gene editing tools are selected based on CGD subtypes. For example, p47phox‐deficient CGD was treated using lentiviral vectors to deliver corrective genes, successfully restoring NADPH oxidase activity [204]. Early γ‐retroviral vectors posed insertional mutagenesis risks (e.g., EVII activation causing myelodysplastic syndrome), whereas self‐inactivating lentiviral vectors (SIN‐LVs) with enhancer deletions enhanced safety [205]. AAV6 has been used to efficiently deliver gene templates, with electroporation enhancing transduction efficiency [203]. Direct delivery of CRISPR ribonucleoprotein (RNP) complexes reduces vector‐related immunogenicity while maintaining cell viability [206]. Additionally, the PE therapy PM359, the first prime editor to enter global Phase 1/2 clinical trials after United States Food and Drug Administration approval (NCT: No. 06559176 /Active, not recruiting/Phase 1/2/Chronic Granulomatous Disease) corrects CYBB or NCF1 mutations in autologous HSCs ex vivo without dsDNA breaks, significantly lowering off‐target risks [207].

#### 5.1.3 Systemic Lupus Erythematosus

Systemic lupus erythematosus (SLE) is a chronic systemic autoimmune disease characterized by immune‐mediated tissue damage and multiorgan inflammation [208]. Because of its complex causes, gene editing remains experimental, focusing on immune regulation, epigenetics, and specific gene targets [209, 210]. Studies highlight the critical role of interferon (IFN) signaling in SLE pathogenesis, with significant upregulation of IFN‐induced genes (e.g., BLK, IL12A) in peripheral blood cells correlating with disease severity [211, 212]. Gene editing can block hyperactive immune responses by knocking down or inhibiting key IFN pathway genes, such as PRDM1 and STAT3 [213]. Additionally, abnormal DNA methylation in SLE lymphocytes drives overexpression of autoimmune genes, which gene editing may correct [210]. Editing B cell master genes like IKZF1 and AIOLOS can suppress aberrant B cell activation and autoantibody production [214]. Knockout of autophagy‐related genes (e.g., Atg5, Atg7) reduces B cell antibody responses or plasma cell differentiation, ameliorating SLE symptoms [215]. Mitochondrial‐associated genes (e.g., IFI27, MSRB2), dysregulated in SLE, can be modulated via gene editing to improve mitochondrial function and attenuate inflammation [216].

#### 5.1.4 Rheumatoid Arthritis

Rheumatoid arthritis (RA) is a chronic inflammatory disorder marked by synovial inflammation, joint destruction, and systemic manifestations [217, 218, 219]. Pathological hallmarks include synovial hyperplasia, immune cell infiltration (e.g., T/B lymphocytes, macrophages), and excessive proinflammatory cytokine secretion [220, 221]. Gene editing strategies target these processes. CRISPR–Cas9 can knock out or disrupt proinflammatory cytokine genes (e.g., TNF‐α, interleukin [IL]‐1) or deliver soluble receptors (e.g., sTNFR, IL‐1Ra) to neutralize their activity [222]. For example, adenoviral delivery of IL‐1Ra reduced synovial fluid IL‐1 levels in a dose‐dependent manner [223]. Inhibiting NF‐κB or JAK–STAT signaling suppresses proinflammatory mediator production [224]. Engineered T cells targeting RA‐associated antigens (e.g., CD20) or secreting anti‐inflammatory factors enable localized immune modulation [225]. Abnormal DNA methylation or histone acetylation in RA may be addressed by inhibiting DNMTs to normalize proinflammatory gene expression [226, 227]. Employing humanized synovial organoids to model the RA microenvironment, Jahid et al. [228] demonstrated that knockout of matrix metalloproteinase genes in fibroblast‐like synoviocytes suppresses their cartilage‐invading capacity. Given RA's polygenic and epigenetic complexity, combination strategies are likely required for efficacy [229].

### 5.2 Applications and Strategies of RNA Editing in Immune System Diseases

RNA editing, a posttranscriptional modification mechanism, offers flexible and safer therapeutic options by altering RNA sequences without permanent genomic changes. Core mechanisms include ADAR‐mediated A‐to‐I editing and APOBEC‐mediated C‐to‐U editing [230]. RNA editing treats immune system diseases by targeting immune checkpoints, modulating inflammatory pathways, and correcting pathogenic mutations [231, 232, 233]. Multiple delivery systems are employed: AAVs offer natural tropism and low immunogenicity but require optimization for cargo capacity and targeting [57]; lentiviruses enable long‐term expression via genomic integration but pose insertional mutagenesis risks [234]; lipid nanoparticles (LNPs), a mature nonviral system, deliver mRNA and CRISPR–Cas9 components with high payload capacity, tunable pharmacokinetics, and avoidance of genomic integration [235, 236]. Other systems include ASOs and RNP complexes.

#### 5.2.1 Severe Combined Immunodeficiency

RNA editing corrects mRNA mutations without genomic integration, offering a new therapy for SCID. X‐SCID, caused by IL2RG mutations disrupting the common γ‐chain (γc) of cytokine receptors, impairs T/NK cell development and B cell function [188]. For IL2RG nonsense mutations (e.g., c.458T>C), A‐to‐I editing may restore the open reading frame to enable γc expression [237]. For missense mutations (e.g., p.R226C), C‐to‐U or A‐to‐I editing could rescue receptor function [188]. In ADA‐SCID, ADAR‐based RNA editing or RNA trans‐splicing can correct G→A mutations, restore ADA expression, and restore metabolic balance [57, 238].

#### 5.2.2 Chronic Granulomatous Disease

In CGD, diverse NADPH oxidase mutations (e.g., missense, nonsense, frameshift) are amenable to RNA editing [199]. For CYBB nonsense mutations (e.g., p.Arg54X), A‐to‐I editing can repair mRNA to restore NADPH oxidase activity [206]. Exon editing (e.g., ADAR‐ or CRISPR–Cas13‐mediated trans‐splicing) corrects splicing defects or frameshifts. Delivery via LNPs or lentiviral vectors to CD34+ HSCs enables lasting effects [239]. Tissue‐specific promoters in targeted vectors may direct editing to bone marrow or peripheral blood cells [239].

#### 5.2.3 Systemic Lupus Erythematosus

SLE pathogenesis involves autoantibody production, overactive IFN signaling, immune complex deposition, and inflammatory mediator dysregulation [240, 241]. RNA editing can modulate IFN pathways by editing 3′UTRs of IFN‐related genes (e.g., IFNAR1) to reduce receptor expression [242]. Engineered ADAR variants (e.g., ADARdd) editing IFN‐α mRNA coding regions reduce IFN‐α secretion and improve nephritis in mouse models [242, 243]. APOBEC3A‐guided RNA editing reduces TLR7/9 ligand‐induced activation, reducing plasmacytoid dendritic cell activation [244]. Editing STAT mRNA to introduce premature stop codons or alter phosphorylation sites (e.g., STAT3 Tyr705) suppresses proinflammatory signaling [245, 246]. B cell modulation via CD19/CD22 mRNA editing reduces activation, while BAFF receptor editing blocks survival signals and autoantibody production [247]. Inhibiting APOBEC3A‐mediated C‐to‐U editing reduces autoantigen generation [244]. Editing Annexin A1 in neutrophil extracellular traps diminishes immunogenicity, ameliorating autoimmune injury [248].

#### 5.2.4 Rheumatoid Arthritis

RNA editing targets RA by suppressing proinflammatory cytokines and immune responses. Editing TNF‐α or IL‐6 mRNA 3′UTRs or coding regions inhibits translation or promotes degradation [57]. ADAR fusion proteins (e.g., REPAIR system) editing IL‐6 receptor mRNA block inflammatory signaling [14]. ADAR1‐mediated viral‐like dsRNA editing prevents MDA5/RIG‐I recognition, reducing IFN release and autoimmune responses [230, 249]. Editing miRNA precursors (e.g., miR‐155) modulates immune cell differentiation and Th17/Treg balance [225]. In patients with RA, RA synovial fibroblasts (RASFs) exhibit aberrant activation, which drives progressive joint destruction [227]. Editing long noncoding RNAs (lncRNAs) (e.g., NEAT1) or miR‐146a in RASFs restores DNA methylation and suppresses invasiveness [250, 251]. Correcting RNA‐modifying single nucleotide polymorphisms in RA susceptibility genes (e.g., PTPN22, HLA‐DRB1) via C‐to‐U editing may reduce disease risk [252].

Targeted RNA editing effectively corrects monogenic mutations in immunodeficiencies and restores immune tolerance in autoimmune diseases by modulating RNA modifications [217]. However, challenges persist. Gene editing requires improved delivery efficiency and reduced immunogenicity, along with advanced delivery systems for precise targeting [65, 218]. RNA editing demands higher efficiency, specificity, and optimized delivery for in vivo stability and durability [219]. Most RNA editing therapies remain preclinical, lacking data on persistence, immune tolerance, and carcinogenic risks [220]. Despite these obstacles, technological refinement, long‐term safety validation, and interdisciplinary integration will position gene and RNA editing as cornerstone approaches in precision immunotherapy. Table 2 summarizes the various applications and strategies of gene editing and RNA editing in the treatment of different neurological diseases.

**TABLE 2:** Therapeutic applications of gene editing and RNA editing in immune system diseases.

Disease category	Medical terminology	Enzyme	Molecular operating principle	Technical implementation strategy	References
Gene‐editing enzyme	Severe combined immunodeficiency	Cas9	SCID‐X1, caused by mutations in IL2RG, may be treated through ex vivo gene correction of HSCs, leveraging their unique ability to repopulate the entire hematopoietic system and restore immune competence.	By using the precise targeting precision of the CRISPR–Cas9 system and the efficient delivery of the AAV6 vector, the normal IL2RG cDNA sequence was integrated into the initiation codon site of the corresponding gene in CD34+ long‐term hematopoietic stem cells (LT‐HSCs) derived from patients, thereby correcting X‐linked severe combined immunodeficiency (SCID‐X1).	[253]
Gene‐editing enzyme	Chronic granulomatous disease	Cas9, ZFN	In chronic granulomatous disease (CGD), mutations in genes encoding components of the NADPH oxidase complex lead to defective reactive oxygen species (ROS) generation, thereby impairing immune cell function. Distinct subtypes are associated with specific genetic defects, including X‐linked mutations in the CYBB gene (encoding gp91phox) and autosomal recessive mutations in NCF1, NCF2, and NCF4. Therapeutic strategies primarily involve gene editing approaches to correct these mutations and restore NADPH oxidase activity.	Using zinc finger nucleases (ZFNs) or CRISPR/Cas9‐mediated homology‐directed repair (HDR) combined with AAV6‐delivered donor templates, functional genes (e.g., CYBB) were site‐specifically integrated into safe‐harbor sites of CD34+ hematopoietic stem cells (HSCs). Alternatively, CRISPR/Cas9D10A was employed to correct CYBB mutations in induced pluripotent stem cells (iPSCs), restoring phagocyte function postdifferentiation.	[203, 206]
Gene‐editing enzyme	Systemic lupus erythematosus	Cas9	In healthy B cells, receptor editing of light chain genes (e.g., Vκ genes) prevents autoreactivity, whereas B cells from systemic lupus erythematosus (SLE) patients exhibit defective receptor editing, failing to efficiently eliminate autoreactive antibodies. Additionally, aberrant DNA methylation and disrupted histone modifications in both T and B cells drive the overproduction of proinflammatory cytokines.	CRISPR/Cas9‐mediated knockdown or suppression of key IFN pathway genes (e.g., PRDM1, STAT3) blocks hyperactivated immune responses, while correction of B cell master genes (IKZF1, AIOLOS) suppresses their aberrant activation and reduces autoantibody production.	[213, 214]
Gene‐editing enzyme	Rheumatoid arthritis	Cas9	Knockout or silencing of proinflammatory genes (e.g., miR‐155) reduces the production of inflammatory cytokines such as TNF‐α, IL‐1, and IL‐6, while activation of apoptotic pathways through gene editing (e.g., galectin‐binding protein‐induced T cell apoptosis) suppresses aberrant proliferation and invasiveness of fibroblast‐like synoviocytes.	CRISPR/Cas9 technology was used to create a miR‐155 gene knockout RAW264.7 macrophage cell line, which exhibited upregulation of Src homology 2 domain‐containing inositol 5‐phosphatase 1 (SHIP1) and a marked reduction in proinflammatory cytokine production. However, treatment with receptor activator of nuclear factor‐κB ligand (RANKL) induced a modest increase in osteoclastogenesis. Parallel CRISPR–Cas9 editing of PTPN2 and PTPN22 genes in Jurkat T cells showed their regulatory roles in immune and inflammatory responses.	[254, 255]
Gene‐editing enzyme	Inflammatory bowel diseases	Cas9	Targeted editing of proinflammatory cytokine genes (e.g., IL12B, TNF) or signaling genes (e.g., NFKB1, JAK–STAT) attenuates inflammatory responses, while genetic modulation of genes involved in differentiation and activation (IL2RA, NOD2) in immune cells (e.g., T cells, macrophages) suppresses excessive immune activation.	Cationic poly beta‐amino ester (PBAE) nanoparticles were complexed with plasmid DNA encoding a destabilized Cas9 nuclease (dsCas9). A biomimetic cell membrane coating was applied to the PBAE/dsCas9 complexes to enable targeted delivery to inflammatory foci. The ROS‐responsive nanocomplexes were activated by elevated reactive oxygen species (ROS) signals at disease sites, thereby stabilizing dsCas9 expression and triggering its genome‐editing activity. This system enabled precise CRISPR/Cas9‐mediated editing of immunomodulatory genes (e.g., Gatm, NOD2, ATG16L1, KRT12) within adaptive immune pathways to suppress pathological inflammation.	[256]
RNA‐editing enzyme	Severe combined immunodeficiency	Cas13, ADAR	RNA editing corrects pathogenic mutations underlying SCID, such as defects in the IL2RG gene, by modifying mRNA sequences to restore the functionality of critical immune proteins (e.g., the common cytokine receptor γ‐chain).	The ADAR deaminase domain (e.g., ADAR1/2) was fused to a deactivated Cas13 (dCas13) protein, enabling A‐to‐I editing at specific mRNA sites guided by sgRNA to correct pathogenic IL2RG mutations. For the c.458T>C mutation in IL2RG that introduces a premature termination codon (PTC), ADAR‐mediated A‐to‐I editing may restore the open reading frame (ORF), thereby rescuing normal γc chain expression.	[237, 257]
RNA‐editing enzyme	Chronic granulomatous disease	ADAR	In CGD, mutations in genes encoding NADPH oxidase components lead to defective ROS generation in phagocytes. Adenosine deaminase acting on ADAR‐mediated A‐to‐I RNA editing corrects these mutations at the transcript level, restoring functional protein expression.	For missense mutations in the CYBB gene (e.g., p.Arg54X), the functional restoration of NADPH oxidase can be achieved through A‐to‐I editing‐mediated mRNA repair.	[206]
RNA‐editing enzyme	Systemic lupus erythematosus	ADAR, APOBEC	In patients with SLE, the IFN pathway is hyperactivated, and signaling pathways such as JAK–STAT exhibit abnormalities. RNA editing holds the potential to modulate the aberrant inflammatory response.	Utilizing the engineered variant ADARdd of ADAR1, targeted editing of the coding region of IFN‐α mRNA was carried out. This approach effectively reduced the secretion of IFN‐α, thereby ameliorating the nephritis symptoms in the murine SLE model. Regarding the JAK/STAT signaling pathway, APOBEC can be harnessed to introduce premature termination codons into STAT mRNA. Alternatively, ADAR editing can be utilized to modify phosphorylation sites such as Tyr705 of STAT3, thus suppressing downstream proinflammatory signals.	[243, 245, 246]
RNA‐editing enzyme	Rheumatoid arthritis	ADAR, Cas13	In patients with RA, an elevated inflammatory state is observed, accompanied by the abnormal activation of synovial fibroblasts (RASF). Aberrantly expressed miRNAs, including miR‐146a and miR‐155, regulate inflammatory and immune responses in RA through the regulation of inflammatory signaling pathways.	The REPAIR system harnesses the catalytically inactive Cas13 (dCas13) to steer the activity of ADAR2, thereby facilitating A‐to‐I editing. This editing mechanism can target and edit IL‐6 receptor mRNA, effectively blocking inflammatory signals. Regarding ADAR1, editing of dsRNA inhibits MDA5/RIG‐I recognition and reduces interferon release. This action may play a role in mitigating the autoimmune response.	[14, 249]
RNA‐editing enzyme	Sjögren's syndrome	ADAR	Immune abnormalities cause gland inflammation and dysfunction. The AQP5 gene, which is closely associated with salivary gland function, may experience dysregulation due to mutations or abnormal regulatory mechanisms. Targeted editing of its mRNA by ADAR has the potential to restore protein functionality.	ADAR1 suppresses the activation of pattern recognition receptors (PRRs), such as MDA5, through A‐to‐I editing of dsRNA, thereby attenuating excessive IFN production and mitigating autoimmune inflammation. Additionally, ADAR enzymes modulate the functionality of microRNA precursors (e.g., miR‐661) by editing their sequences, which directly alters the target specificity of the mature microRNAs. This posttranscriptional modification subsequently regulates the expression of inflammatory mediators, including IL‐6 and TNF‐α.	[258]

## 6 Applications of Gene Editing and RNA Editing in Cancer Therapy

Cancer is a major global health problem and the second leading cause of death [259]. Traditional treatment methods, such as surgery, radiotherapy, and chemotherapy, are effective to some extent but are often accompanied by severe side effects and drug resistance problems [260, 261]. In recent years, the rapid development of gene editing and RNA editing technologies has provided new strategies for cancer treatment. These technologies, by precisely modifying genes or RNA in cancer cells, can inhibit tumor growth, boost the immune response against cancer, and lessen treatment side effects [262, 263].

### 6.1 Application of Gene Editing in Cancer Therapy

The application and strategies of gene‐editing technologies in cancer treatment have become a research hotspot in the field of biomedical science. In recent years, with the rapid development of gene‐editing technologies such as CRISPR–Cas9, their potential in cancer treatment has gradually been explored.

#### 6.1.1 Direct Targeting of Cancer Cell Genes

Gene editing played an important role in the repair of tumor suppressor genes. CRISPR technology exerted influence by correcting the gene mutation of tumor suppressor gene, DNA repair gene or other driving genes, so as to repair the mutated tumor suppressor gene and restore its normal tumor suppressor function. For example, in models of liver cancer, breast cancer, and prostate cancer, repairing tumor suppressor genes such as TP53 and PTEN might inhibit tumor growth [264, 265, 266, 267, 268]. CRISPR‐mediated repair of BRCA1 inhibits cancer cell growth and increases chemosensitivity, which is significant for ovarian cancer treatment.

At the same time, gene editing technology could directly knock out oncogenes (such as MYCN, KRAS, BCR–ABL fusion genes) and block the proliferation of cancer cells [269]. The abnormal activation of these genes was a key factor in the occurrence and progress of quantities of cancers. Knocking out these oncogenes by CRISPR technology could block the proliferation signal pathway of cancer cells, thus inhibiting the growth of tumors. For example, in pancreatic cancer cells, the proliferation ability of tumor cells decreased significantly after the KRAS gene was knocked out by CRISPR technology [270]. Targeting MYCN‐amplified neuroblastoma with CRISPR–Cas9 blocked neuronal differentiation and inhibited tumor growth in vitro and in vivo [271].

Additionally, gene editing technology could not only directly modify gene sequences, but also affect gene expression by regulating epigenetic modification. For example, by editing the promoter or enhancer region, silenced tumor suppressor genes could be activated or oncogene expression could be inhibited [272].

#### 6.1.2 Enhance the Effect of Immunotherapy

Gene editing was able to participate in the optimization of chimeric antigen receptor (CAR)‐T cells, transform immune cells and enhance their antitumor ability. CAR‐T therapy is an immunotherapy that engineers T cells to express CAR. These immune cells had the ability to specifically recognize and attach to protein on the surface of tumor cells. This process activated T cells and enhanced their antitumor activity, which eventually contributed to the elimination of cells expressing antigens [273]. Knocking out immune checkpoint molecules (e.g., PD‐1, CTLA‐4) by gene editing technology could enhance the antitumor activity of T cells. Meanwhile, inserting CAR sequences targeting tumor antigens was able to effectively identify and attack cancer cells, thus improved the specificity of CAR‐T cells. Recently, a conductive‐hydrogel electroporation system directly delivered CRISPR–Cas9 targeting PD‐1 into lymph node T cells, producing PD‐1‐deficient T cells that resisted tumor growth, metastasis, and recurrence in mouse melanoma models [274]. In addition, by using CRISPR/Cas9 system to destroy multiple genomic sites at the same time, universal CAR‐T cells lacking the expression of endogenous TCR and HLA class I (HLA‐I) were produced. These biallelically deficient T cells are less sensitive to allogeneic reaction and had the potential to prevent GVHD [275].

#### 6.1.3 Targeting Tumor Microenvironment

Cancer‐associated fibroblasts (CAFs) promote tumor progression in the tumor microenvironment. They had the ability to secrete a variety of cytokines and growth factors, which promoted the proliferation and invasion of tumor cells [276]. Through gene editing technology, CAFs could be engineered to suppress their tumor‐promoting functions [277]. Angiogenesis in the tumor microenvironment was one of the key factors for tumor growth and metastasis. By using gene editing technology to target vascular endothelial growth factor (VEGF) and its receptor (VEGFR), the blood supply to tumors was able to be inhibited. For example, using CRISPR technology to knock out the VEGF gene in tumor cells or the tumor microenvironment could reduce tumor angiogenesis, thereby inhibiting tumor growth [278, 279]. The extracellular matrix (ECM) played important structural and functional role in the tumor microenvironment. Abnormal changes in the ECM contributed to tumor invasion and metastasis [280]. Through gene editing technology, key proteins in the ECM, such as focal adhesion kinase, could be targeted to inhibit the activity of abnormal changes, thereby reducing tumor invasion and metastasis [281]. Metabolic reprogramming in the microenvironment drives tumor growth and immune escape. Gene editing technology took effect on targeting metabolic pathways in the tumor microenvironment to alter their metabolic status [282]. By using CRISPR technology to knock out metabolic‐related genes in tumor cells (such as LDHA), the metabolic products in the tumor microenvironment could be changed, thereby inhibiting tumor growth [283].

#### 6.1.4 Cancer Model Construction and Drug Screening

Gene editing technology had the ability to accurately introduce specific gene mutations into cell or animal models and simulate the occurrence and development of human cancer. For example, CRISPR‐mediated knock‐in or knock‐out of oncogenes or tumor suppressors in cell lines creates in vitro models that closely resemble human cancers [284, 285, 286]. These models could be used to study the pathogenesis of cancer, the biological behavior of tumor cells and the mechanism of drug action. Using gene editing technology, a series of homologous cell line models have been constructed. These lines share the same genetic background but carry different mutations. For example, knocking in different mutations in the same cell line by CRISPR technology was beneficial to study their effects on tumor cell phenotype. [287, 288].

Gene editing combined with high‐throughput screening was used to rapidly identify potential drug targets. For example, genome‐wide knockout screening of cancer cells by CRISPR–Cas9 system identified genes essential for tumor cell growth and survival [288, 289, 290]. These genes could be used as potential drug targets and provide basis for developing new anticancer drugs. CRISPR–Cas9 screening of tumor suppressors in colorectal cancer organoids predicts patient response to TGF‐β inhibitors [291]. Gene editing technology could also be applied to study the drug resistance mechanism of tumor cells. Introducing specific gene mutations into cancer cells helps simulate clinical drug resistance and assess its impact on drug sensitivity. For example, using CRISPR technology to introduce mutations related to drug resistance into cell models in vitro was beneficial to quickly identify the key genes leading to drug resistance and provide strategies for overcoming drug resistance [292, 293, 294]. Based on the above characteristics, gene editing technology was able to build a personalized cancer model according to the patient's specific gene mutation, which could be used to screen drugs suitable for patients.

### 6.2 Application of RNA Editing in Cancer Therapy

RNA editing was a technique to regulate gene expression by changing RNA sequence. Different from gene editing, RNA editing did not change the DNA sequence, but affected the synthesis and function of protein by modifying RNA molecules. In recent years, RNA editing has shown potential application value in the field of cancer treatment because of its high specificity and reversibility.

#### 6.2.1 Correction of Oncogene Mutation

RNA editing technology targeted and edited the RNA of oncogenes (such as point mutations and fusion genes), directly repairing erroneous sequences to prevent the production of oncogenic proteins, without directly modifying the genomic DNA [295]. This reversible and transient editing approach avoided the potential permanent risks associated with DNA editing. ADAR expression was associated with various cancers. RNA editing of antienzyme inhibitor 1 (AZIN1) has been shown to promote malignant progression of esophageal squamous cell carcinoma, hepatocellular carcinoma, non‐small cell lung cancer, and colorectal cancer [296, 297, 298, 299, 300]. On the one hand, RNA editing has been proved to change serine into glycine at position 367 of AZIN1 protein, which led to protein conformation change, protecting ornithine decarboxylase and cyclin D1 from degradation, and thus promoted tumor cell proliferation. On the other hand, AZIN1 protein edited by RNA played a key role in numerous malignant tumors by upregulating IL‐8 and promoting angiogenesis [41]. In endometrial carcinoma, the overexpression of ADAR1‐mediated RNA editing of AZIN1 was related to poor prognosis [301]. ADAR1‐dependent RNA editing of CDK13 was found to promote the progress of thyroid cancer [302].

However, RNA editing could also inhibit the proliferation of tumor cells by affecting gene expression or signal pathway. In non‐small cell lung cancer, mutations in the EGFR gene were common. Modulated RNA editing repaired the mutated EGFR transcripts, thereby inhibiting tumor growth [303]. In hepatocellular carcinoma, ADAR2‐catalyzed COPA^I164V^ mutation was able to inhibit PI3K/AKT/mTOR signaling pathway, thus exerting tumor inhibition effect [304].

#### 6.2.2 Targeting Noncoding RNA

Noncoding RNAs (ncRNAs) are RNA molecules that do not encode proteins, including microRNAs (miRNAs), lncRNAs, circRNAs, and PIWI‐interacting RNAs, which play crucial roles in cancer initiation and progression [305].

miRNA mainly targeted 3′UTR of mRNA to induce degradation or inhibit translation. RNA editing exerted influence on affecting the combination of miRNA and mRNA by changing the recognition sequence of miRNA, splicing mode and miRNA maturation, thus leading to tumor progression [306]. Current studies show that delivering miRNA mimics can inhibit metastasis and stemness in various cancers. The systemic delivery of tumor suppressor factors miR‐34a or miR‐143/145 using nanocarriers effectively inhibited the growth of pancreatic cancer in mice [307]. The delivery of miRNA‐34a mediated by chitosan nanoparticles contributed to autophagy, thus inhibiting the growth of prostate tumor in bone [308]. Polyarginine‐disulfide bond‐conjugated polyethylenimine nanocarriers for the delivery of miR‐145 significantly inhibited prostate tumor growth and prolonged survival time [309].

RNA editing could change the secondary structure of lncRNA and then affect its interaction with protein or other RNA molecules. LncRNA played a significant role in cancer through promoter loop, alternative splicing, antisense gene silencing, transcription regulation, and DNA repair [310]. Loss of A‐to‐I editing in lncRNA GAPLINC promotes release of oncogenic miRNAs (e.g., miR‐331‐3p) in GBM [311]. In addition, the lncRNA MEG3 was highly expressed in the normal cerebral cortex, but its expression was reduced or absent in virous cancers. Reintroducing lncRNA MEG3 into GSCs was able to inhibit cell proliferation and tumor growth in vivo [312].

#### 6.2.3 Early Diagnosis and Prognosis Evaluation

RNA editing databases could be utilized for bioinformatics searches to identify RNA editing sites at specific genomic locations, which might aid in the detection of early‐stage tumors, thus potentially linking them to the process of early cancer diagnosis and prognosis evaluation [313]. Gabra3 specific RNA editing sites (such as I342M) have been shown to detect breast cancer [314]. ADAR2‐mediated RNA editing PODXL gene H241R site could be applied to detect gastric cancer [315]. In addition, RNA editing at S367G site of Azin1 gene influences detection of hepatocellular carcinoma, esophageal squamous cell carcinoma, and colorectal cancer [296, 316].

The level of RNA editing closely correlates with patient prognosis and serves as a key marker of disease progression and treatment response. Multiple studies have explored RNA editing as a prognostic indicator for cancer. The results have shown that RNA editing signatures was beneficial to stratify patients with gastric cancer and esophageal squamous cell carcinoma based on their potential benefits from chemotherapy [317, 318]. In addition, the expression levels of ADAR2 in GBM, ADAR1 in melanoma, and ADAR2 in hepatocellular carcinoma were all closely related to the clinical staging and patient survival rates of these malignancies [319, 320, 321].

#### 6.2.4 Reversal of Drug Resistance

RNA editing's role in tumor drug resistance is an important focus in cancer research and therapy. ADAR1 played a crucial role in the progression of non‐small cell lung cancer and was associated with the sensitivity of cancer cells to anticancer drugs. Knocking down the expression of the ADAR1 gene resulted in the inhibition of cell proliferation, cell cycle arrest, and apoptosis, subsequently increasing the sensitivity of non‐small cell lung cancer cells to anlotinib [322]. TRAF6 overexpression may result from loss of miR‐146b‐5p regulation due to excessive RNA editing, increasing GBM cells’ resistance to temozolomide [323].

### 6.3 Future Prospects in Cancer Therapy

Gene editing technology, particularly the CRISPR/Cas9 system, holds tremendous promise for cancer treatment. The future research direction mainly focuses on the following aspects [324]. First, optimize editing tools and develop a safer and more effective delivery system to improve specificity and efficiency and reduce off‐target effect. Second, develop new gene editing tools, such as base editor and PE, to achieve more accurate gene repair. Third, combined with the genomic characteristics of tumor cells in patients, attention should be focused on the individualized treatment. Additionally, combine gene editing technology with other treatment methods (such as chemotherapy and immunotherapy) to enhance the antitumor effect.

The future research direction of RNA editing in cancer treatment mainly includes the following aspects [41]. First, develop more efficient RNA editing tools that are able to improve editing efficiency and specificity, and reduce off‐target effect. Second, RNA editing technology is used to correct the mutation of oncogene, and normal gene function can be restored by regulating cells and molecules in tumor microenvironment, thus inhibiting tumor growth. Further explore the potential of RNA editing as a biomarker for early diagnosis and prognosis evaluation of cancer. Combine RNA editing with other therapies to enhance antitumor responses. Table 3 summarizes the future directions of gene and RNA editing in cancer.

**TABLE 3:** Future directions of gene and RNA editing in cancer.

Future direction	Gene editing	RNA editing
Technology optimization	Develop highly specific Cas variants to reduce off‐target effects Improve tumor‐targeted delivery [325]	Enhance ADAR enzyme efficiency Develop light‐controlled RNA editors for spatiotemporal precision [5]
Oncogene/tumor suppressor targeting	Permanent knockout of driver mutations Restore tumor suppressor gene function [269]	Reversible suppression of oncogenic mRNA translation Correct aberrant splicing in tumor suppressors [295]
Immunotherapy enhancement	CRISPR‐engineered CAR‐T cells to boost antitumor activity [273]	Temporarily modulate immune checkpoint molecules [326]
Overcoming drug resistance	Edit cancer stem cell genes [285] Reverse chemotherapy resistance [288]	Transient modulation of drug‐metabolizing enzymes Block resistance pathways [327]
Combination therapies	Gene editing can be combined with other cancer treatments such as chemotherapy, radiation therapy, or immunotherapy to enhance overall therapeutic efficacy [324]	RNA editing can be used in combination with other therapies. By modulating gene expression at the RNA level, it can synergize with existing treatments to achieve better outcomes [328]
Personalized cancer treatment	Gene editing can be tailored to target specific mutations or genetic alterations in individual patients’ tumors, providing personalized treatment strategies [324]	RNA editing can be personalized based on the specific genetic and molecular characteristics of a patient's cancer, to modify the expression of genes unique to the tumor, improving the precision and effectiveness of treatment [329]
Delivery systems	Tumor‐specific viral vectors (e.g., oncolytic viruses with CRISPR) Nanoparticle‐based targeting [330]	Exosome‐mediated RNA editor delivery Degradable LNPs for transient regulation [331]

## 7 Comparison Between Gene Editing and RNA Editing

Gene editing and RNA editing are two distinct gene regulation technologies that operate at the DNA and RNA levels, respectively. They exhibit significant differences in mechanisms, applications, safety, advantages, and disadvantages. A comprehensive comparison is provided below.

### 7.1 Comparison of Technical Principle and Operation Process

Gene editing technology, especially CRISPR–Cas9, offers unparalleled accuracy and efficiency in correcting gene mutations. Gene editing technology, especially the CRISPR–Cas9 system, uses a gRNA to direct the Cas9 protein to specifically recognize and cleave the target DNA, generating a DSB. The cell then repaired the break through NHEJ or HDR, thereby achieving gene knockout, knock‐in, or point mutation. The workflow mainly included the following steps: designing a specific gRNA, cloning it together with the Cas9 gene into a plasmid; transfecting the plasmid into target cells; and screening for successfully edited cells through PCR and sequencing, followed by functional analysis.

RNA editing provided a flexible and reversible approach to modulate gene expression and protein function without altering the genome. RNA editing technology modifies RNA molecules through specific enzymes (such as ADAR enzymes) or artificially designed editing systems (such as PE for RNA), thereby regulating gene expression or correcting mutations without directly modifying the genomic DNA. The workflow mainly included the following steps: designing a gRNA (such as sgRNA or eagRNA) that was complementary to the target RNA sequence, and constructing it with the editing enzyme (such as ADAR2) or editing system into an expression vector; delivering the vector into target cells via liposome‐mediated transfection or viral vector delivery; and verifying the efficiency and specificity of RNA editing through RNA sequencing and other methods.

### 7.2 Comparison of Applications

DNA editing and RNA editing have distinct therapeutic applications. DNA editing is primarily used for diseases requiring long‐term or permanent genetic correction, such as monogenic disorders [332], inherited immunodeficiencies [239], and certain cancer gene therapies [271]. Its key advantage lies in its ability to fundamentally repair disease‐causing mutations, though it carries potential genomic safety risks. In contrast, RNA editing is better suited for conditions requiring dynamic regulation or short‐term intervention, including neurodegenerative diseases (AD, PD) [130, 132], acute inflammatory [333] or metabolic disorders [334], and certain RNA‐mediated genetic diseases (e.g., specific types of ALS) [335]. RNA editing is characterized by its reversibility and absence of genomic integration risks, but requires repeated administration to sustain therapeutic effects.

### 7.3 Comparison of Safety and Effectiveness

Gene editing can directly modify DNA sequences, targeting specific DNA sequences with high specificity, thereby correcting pathogenic mutations at the source to produce permanent effects. This makes it suitable for addressing genetic variations or hereditary diseases that required long‐term solutions. The application scope of gene editing is broad, covering genetic improvement in animals and plants as well as the treatment of human diseases [336, 337, 338]. Additionally, the CRISPR–Cas9 system, by designing specific sgRNAs, was able to precisely edit target genes, including gene knockouts, knock‐ins, and point mutations. Its high editing efficiency could be further enhanced through optimization of sgRNA design and delivery methods [339]. However, gene editing carried a certain risk of off‐target effects, which resulted in the unintended mutations at nontarget sites. Studies have shown that the off‐target frequency might be ≥50% [340]. The occurrence of off‐target effects was related to factors such as the sequence design of sgRNA, the activity of Cas proteins, and the cellular environment [341]. To reduce off‐target risks, researchers have developed various strategies, such as using truncated sgRNAs, chemically modified sgRNAs, RNP delivery formats, and the development of high‐fidelity Cas9 variants [342].

RNA editing did not alter the genome but dynamically adjusted RNA sequences posttranscriptionally. Its reversibility and flexibility provide unique advantages in certain application scenarios [341]. RNA editing had unique advantages in the treatment of certain neurodegenerative diseases, as temporal and spatial control of protein function was crucial in these diseases. Since RNA editing could not cause permanent genomic changes, it is generally considered safer than gene editing, thereby avoiding potential genomic instability and long‐term risks [343]. RNA editing did not rely on HDR, meaning it could be used in nonsplinter cells [344].

### 7.4 Comparison of Limitations

One major issue with gene editing was the relatively high frequency of off‐target effects. Another significant problem was that gene editing can only function in proliferating cells, as CRISPR nucleases required the presence of a PAM sequence, which limited the range of targetable sites [345]. Due to the competition between the NHEJ and HDR repair pathways and the differences in repair mechanisms in specific cell types, the editing outcomes might be uncontrollable. Moreover, as gene editing involved direct modification of the genome, its application in human embryos and germline cells has sparked numerous ethical and legal controversies, also restricting its use to some extent [346].

The limitations of RNA editing were also quite evident. First, the effects of RNA editing were temporary, affecting only RNA transcripts, and were reversible, which might not be sufficient in therapeutic scenarios requiring long‐term or permanent gene changes [155]. Second, due to the rapid degradation of RNA molecules, repeated interventions were needed during clinical treatment to maintain efficacy, which could significantly increase the cost of treatment [1]. Another major issue was the limited scope of application for RNA editing. Although RNA editing technologies had unique advantages in terms of safety, they still had limitations in the precise control of endogenous genes. For example, traditional RNA editing technologies could only delete RNA fragments in multiples of six nucleotides, restricting their use in certain application scenarios [347]. Table 4 presents the comparison of each dimension of gene editing and RNA editing.

**TABLE 4:** Comparison of gene editing and RNA editing.

Comparative dimension	Gene editing	RNA editing
Mechanism	Directly modifies DNA (e.g., CRISPR–Cas9 induces double‐strand breaks, repaired via NHEJ/HDR) [348]	Modifies RNA sequences (e.g., ADAR converts A→I or Cas13 cleaves target RNA). No genomic changes [343]
Persistence	Permanent [348]	Transient [343]
Applications	Monogenic disorders [332] Inherited immunodeficiencies [239] Cancer gene therapies [271]	Neurodegenerative diseases [132] Acute inflammatory [333] Metabolic disorders [334] RNA‐mediated genetic diseases [335]
Safety	Off‐target DNA mutations Ethical concerns [346]	Off‐target effects limited to RNA (no genomic risk) Fewer ethical issues [155]
Efficiency	High (long‐lasting effects) Depends on DNA repair pathways (e.g., HDR inefficiency) [348]	Requires repeated dosing (due to RNA turnover) Efficiency depends on editor activity and delivery [349]
Delivery	Viral vectors (AAV, lentivirus) Nanoparticles Electroporation (ex vivo) [25]	LNPs (lipid nanoparticles) AAV Chemically modified RNA (e.g., mRNA vaccines) [331]
Limitations	Irreversible risks Large edits are challenging Poor in vivo delivery efficiency [339]	Short‐lived effects Frequent administration needed Tissue‐specific delivery hurdles [350]
Key technologies	CRISPR–Cas9 Base editing Prime editing [351]	ADAR‐mediated editing CRISPR–Cas13 RESCUE (C→U editing) [352]

### 7.5 Comparison of Developmental Stages

DNA editing and RNA editing show significant differences in developmental stages. DNA editing technology has established a relatively complete R&D pipeline. In the basic research stage, DNA editing primarily focuses on CRISPR system optimization, such as the development of base editing and PE technologies [353]. Preclinical studies of DNA editing emphasize addressing critical issues such as delivery efficiency and off‐target effects [354]. The clinical stage has achieved breakthrough progress—multiple therapies for blood disorders like β‐thalassemia and sickle cell disease have entered clinical trials [281], with Casgevy becoming the first approved CRISPR‐based gene‐editing drug [355]. In contrast, RNA editing technology lags behind in overall R&D progress. Basic research in RNA editing is dedicated to developing novel editing tools like ADAR systems and CRISPR–Cas13 [14]. Preclinical studies mainly tackle challenges such as editing persistence and delivery system optimization [356]. Currently, only a few projects (e.g., Wave Life Sciences’ therapy for alpha‐1 antitrypsin deficiency, ClinicalTrials.gov Identifier: NCT05120830) have entered early‐stage clinical trials, with the field still in the preliminary phases of proof‐of‐concept and safety evaluation.

## 8 Discussion

The synergistic development of gene editing and RNA editing technologies is transforming in disease treatment paradigms. As dual engines of precision medicine, these technologies enable programmed manipulation of genetic information through multitiered interventions: Gene editing, centered on CRISPR–Cas systems, provides lasting cures for genetic disorders through permanent genomic modifications, exemplified by base editing‐mediated repair of HBB mutations in sickle cell anemia achieving functional cure in clinical trials. RNA editing employs ADAR or Cas13 systems to enable reversible regulation of transcripts, creating new therapeutic avenues for dynamic pathological processes. This DNA–RNA two‐axis regulatory strategy not only addresses the full spectrum from genetic defect correction to phenotypic modulation, but also achieves precise expansion of therapeutic windows through spatiotemporal‐specific interventions.

In therapeutic applications, these technologies demonstrate remarkable complementary strengths. Gene editing can overcome the limits of personalized therapies through universal CAR‐T cell engineering while enhancing reversal of drug resistance with PE‐mediated activation of tumor suppressor genes. RNA editing achieves tumor microenvironment reprogramming through immune checkpoint molecule regulation, with its transient activity profile avoiding genomic integration risks and enabling dynamic immune balance through dosage optimization. The “static repair + dynamic regulation” integrated strategy has been validated across multiple disease models, showing that multimodal technological integration could break through efficacy barriers inherent to single‐modality approaches.

Nevertheless, three critical challenges persist. First, current editing precision remains inadequate for complex disease demands, with CRISPR–Cas9 exhibiting off‐target risks during multicopy oncogene editing and RNA editing showing <35% efficiency in lncRNA regulation. Second, delivery systems face the paradox between tissue penetrance and immune compatibility, where LNP carriers demonstrate <0.1% delivery efficiency in brain parenchyma while AAV9‐mediated spinal cord delivery may induce CD8+ T cell infiltration. Third, the theoretical framework for technological synergy remains incomplete, particularly lacking quantitative models to guide combination therapy design considering epigenetics‐posttranscriptional regulation crosstalk.

Future advancements should focus on three dimensions: Technologically, developing tissue‐specific intelligent editors (e.g., optogenetic CRISPR–CasX systems) and self‐regulating RNA editing complexes (e.g., miRNA‐responsive ADAR variants) could enhance safety through closed‐loop control. Mechanistically, establishing multiomics‐guided editing decision systems that integrate single‐cell epigenomic maps with dynamic RNA structural predictions would enable full‐process optimization from target selection to editing strategy. In clinical translation, creating switchable therapeutic platforms—such as employing gene editing to establish disease‐resistant genetic backgrounds (e.g., CCR5Δ32 mimicry) combined with RNA editing for phenotypic fine‐tuning—could develop hierarchical intervention strategies that mitigate off‐target risks while adapting to disease heterogeneity. Through cross‐scale technological integration and full‐cycle risk management, the coordinated application of gene and RNA editing promises to inaugurate a new era extending from disease treatment to physiological enhancement.

## Author Contributions

Jia‐Mei Li, Jie Huang, and Yan Liao: conceptualization and writing—original draft. Jie Huang, Yan Liao, and Ting Hu: writing—review. Chang‐Li Wang, Chen‐Wei Huang, and Wangzheqi Zhang: supervision and funding acquisition. All authors have read and approved the final manuscript.

## Ethics Statement

The authors have nothing to report.

## Conflicts of Interest

The authors declare no conflicts of interest.

## Data Availability

All data are freely available from the corresponding author upon request.
